# Microbiological insights into anaerobic digestion for biogas, hydrogen or volatile fatty acids (VFAs): a review

**DOI:** 10.1080/21655979.2022.2035986

**Published:** 2022-02-25

**Authors:** Sharareh Harirchi, Steven Wainaina, Taner Sar, Seyed Ali Nojoumi, Milad Parchami, Mohsen Parchami, Sunita Varjani, Samir Kumar Khanal, Jonathan Wong, Mukesh Kumar Awasthi, Mohammad J. Taherzadeh

**Affiliations:** a Swedish Centre for Resource Recovery, University of Borås, 50190 Borås, Sweden; b Microbiology Research Center, Pasteur Institute of Iran, Tehran, Iran; c Department of Mycobacteriology and Pulmonary Research, Pasteur Institute of Iran, Tehran, Iran; d Paryavaran Bhavan, Gujarat Pollution Control Board, Gandhinagar, Gujarat, India; e Department of Molecular Biosciences and Bioengineering, University of Hawaii at Manoa, Honolulu, Hawaii, USA; f Department of Biology, Institute of Bioresource and Agriculture and, Hong Kong Baptist University, Hong Kong; g College of Natural Resources and Environment, Northwest A&F University, Taicheng Road 3#, Yangling, Shaanxi, 712100, China

**Keywords:** Anaerobic digestion, artificial rumen, interspecies electron transfer, microbial communities, syntrophy, Wood-Ljungdahl pathway

## Abstract

In the past decades, considerable attention has been directed toward anaerobic digestion (AD), which is an effective biological process for converting diverse organic wastes into biogas, volatile fatty acids (VFAs), biohydrogen, etc. The microbial bioprocessing takes part during AD is of substantial significance, and one of the crucial approaches for the deep and adequate understanding and manipulating it toward different products is process microbiology. Due to highly complexity of AD microbiome, it is critically important to study the involved microorganisms in AD. In recent years, in addition to traditional methods, novel molecular techniques and meta-omics approaches have been developed which provide accurate details about microbial communities involved AD. Better understanding of process microbiomes could guide us in identifying and controlling various factors in both improving the AD process and diverting metabolic pathway toward production of selective bio-products. This review covers various platforms of AD process that results in different final products from microbiological point of view. The review also highlights distinctive interactions occurring among microbial communities. Furthermore, assessment of these communities existing in the anaerobic digesters is discussed to provide more insights into their structure, dynamics, and metabolic pathways. Moreover, the important factors affecting microbial communities in each platform of AD are highlighted. Finally, the review provides some recent applications of AD for the production of novel bio-products and deals with challenges and future perspectives of AD.

## 1. Introduction

**Figure 1.**
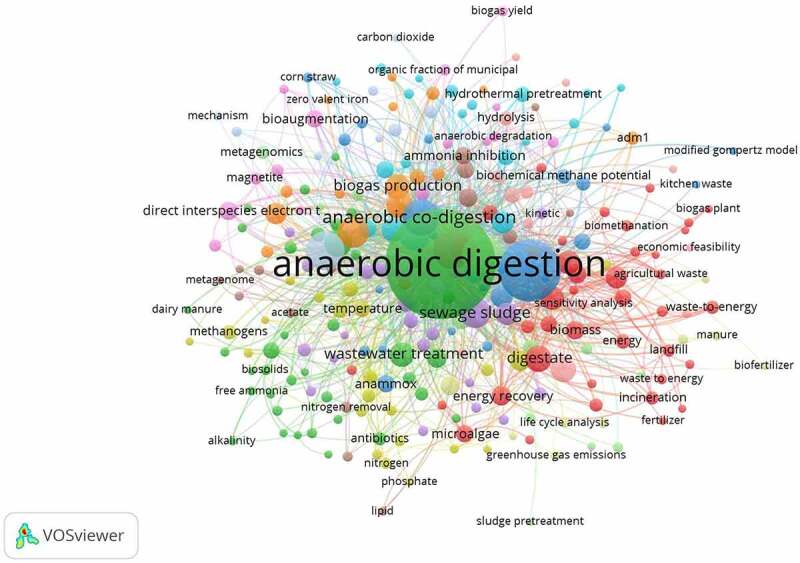
The bibliometric mapping of anaerobic digestion in 2021–2022.

The dependence of the world community on non-renewable energy resources to maintain quality of life, sustain economic development and to enable our vast transportation network is perhaps one of the most important problems facing the world today. Dwindling reserves with rapidly increasing consumption rates, combined with unstable energy prices and the environmental concerns, especially climate change demand that there is an urgent need to develop a sustainable, affordable, and environmentally friendly energy resources. Moreover, world population growth and rapid industrialization resulted in the generation of enormous wastes such as food waste, solid municipal waste, organic waste, agricultural residues, etc. that expected to increase to 70% by 2050. These wastes can be valorized into renewable energy sources and chemicals through different technologies including biological approaches [1,2]. Anaerobic digestion (AD) is one of the most promising alternatives to non-renewable energy resources [3]. To visualize recent distinguished work on AD, various databases were explored herein to acquire the suitable publications and data regarding this topic in 2021–2022 (Figure 1).

AD – a process in which organic materials are digested in the absence of molecular oxygen – has been employed widely for various purposes such as biogas production, waste management, and pathogen deactivation among others. In the natural environments, where oxygen content is limited, such as landfills, sediments, waterlogged soils, or intestinal tracts of ruminants, anaerobic environment exist [4,5]. Assyrians were the first people who employed AD to warm bathwater. In the 16th century, Persians also used AD to heat water. In the 17th century, Alessandro Volta recognized that anaerobic degradation of organic compounds could produce flammable gases such as methane during his summer trip to Lake Maggiore. However, until the 1880s, AD was not used in a full-scale application. In 1895, AD was employed in a hybrid system to treat wastewater of the city of Exeter in the United Kingdom. Two years later, in 1897, digestion plant was built up in Matunga, India, with biogas collection system. Afterwards, in the 20th century, a two-stage system (Travis Tank) was developed and subsequently this process was widely used to treat wastewaters, municipal solid wastes, sewage sludge, manures, industrial organic wastes, etc. [6,7]. During the 1930s, microbiologists made considerable efforts to understand the mechanisms of biogas production in the anaerobic digesters. However, the application of the AD process was limited until 1950 due to the lack of understanding of the fundamentals of AD. With a better understanding of the AD process, various small and industrial applications were developed [7]. Moreover, developments of modern techniques as well as technical equipment and also advances in the recovery of produced gases have reduced the total cost resulting in broad applications of AD.

The AD process provides a precisely balanced ecological environment that successive break down of the organic macromolecules, i.e. carbohydrates, proteins, and lipids to soluble organics, which are subsequently converted into biogas by diverse groups of bacteria and archaea in the absence of oxygen [8–10]. These microorganisms work interactively in four complicated and interdependent biochemical reactions, namely hydrolysis, acidogenesis, acetogenesis, and methanogenesis that resulting in the degradation of organic materials and production of H_2_, CO_2_ and CH_4_ as well as H_2_S in trace amounts [11–14]. In Table 1, some examples of various microorganisms involved in the different AD phases are shown. AD is traditionally used for waste treatment and bioenergy production, but it is being developed as a new platform in which other bio-products can be produced. However, several microbiological and operational challenges need to be resolved to achieve feasible platforms of the AD process for various final products. Extensively, microbiological aspects of AD process may differ from other industrial processes. Because in this process, sterility or pure cultures might not be necessary, while novel microbiological procedures or techniques such as whole-genome sequencing (WGS), next-generation sequencing (NGS), comparative analysis, microcosms studies, and omics approaches such as genomics, metagenomics, transcriptomics, metatranscriptomics, proteomics, metaproteomics, metabolomics, or meta-metabolomics can be more effective and applicable [15–17].

**Table 1.** Microorganisms involved in the various phases of the AD process [4,6,13,25,41,55]

AD Phase	Microbial Domain	Microbial Genus	Examples of Identified Species
Hydrolysis and Fermentation	Bacteria	*Acetivibrio, Aminobacterium, Aminomonas, Anaeromusa, Anaerosphaera* *Bacillus, Bacteroides, Bifidobacterium, Butyrivibrio* *Caldanaerobacter, Caldicellulosiruptor, Campylobacter, Cellulomonas, Clostridium* *Devosia* *Espiroquetas. Eubacterium* *Fervidobacterium, Fibrobacter, Fusobacterium* *Gelria, Gracilibacter* *Halocella* *Lactobacillus* *Paludibacter, Peptococcus, Peptoniphilus, Proteiniborus, Pseudomonas, Psychrobacter* *Ralstonia, Ruminoclostridium, Ruminococcus* *Selenomonas, Shewanella, Sporanaerobacter, Streptococcus, Streptomyces* *Thermanaerovibrio, Thermomonas, Thermomonospora, Thermotoga, Treponema, Trichococcus*	*Pseudomonas mendocina* *Bacillus halodurans* *Clostridium hastiforme* *Gracilibacter thermotolerans* *Thermomonas haemolytica*
	Fungi	*Aspergillus* *Humicola* *Penicillium* *Trichoderma*	*Trichoderma reesei*
Acetogenesis	Bacteria	*Acetobacterium* *Clostridium* *Desulfotignum* *Eubacterium* *Holophaga* *Moorella* *Ruminococcus* *Sporomusa* *Thermoanaerobacter, Treponema*	*Moorella thermoacetica* *Desulfotignum phosphitoxidans* *Holophaga foetida*
Methanogenesis	Archaea	*Methanobacterium, Methanobrevibacter, Methanococcus, Methanoculleus, Methanosaeta, Methanomicrobium, Methanosarcina, Methanospirillum, Methanothermobacter*	*Methanobrevibacter smithii* *Methanobrevibacter arboriphilus* *Methanococcus vannielii*

Microbial diversity of anaerobic digesters depends on various factors such as feedstock type, seed inoculum, temperature, granulation, aeration, mixing speed, pre-treatment type, digester design, organic loading rate (OLR), solids retention time (SRT) and hydraulic retention time (HRT). For example, by increasing the temperature, the diversity of archaeal and bacterial communities decreases considerably. Moreover, short HRT and high OLR are related to *Acidobacteria* community while long HRT and low OLR are associated with *Planctomycetes, Actinobacteria*, and *Alcaligenaceae* [13,18,19]. Overall, most known microbial communities in the anaerobic digesters are prokaryotic ones, while some eukaryotic microorganisms take part in the digestion process such as fungi and protozoa. Anaerobic fungi (particularly the phylum *Neocallimastigomycota*) found in the microbial communities are responsible for the hydrolysis process [13,20–22]. However, these hydrolytic fungi grow slowly, affecting their frequency in the anaerobic digesters. For example, *Neocallimastix* is an anaerobic fungal genus found in the rumen and produces a wide range of hydrolytic enzymes such as xylanase, cellulase, and esterase. The co-culture of this fungus with methanogens resulted in direct methane production from cellulose. Other anaerobic genera involved in the AD of cellulose belong to *Anaeromyces, Caecomyces, Orpinomyces*, and *Piromyces* [4,23,24]. Among prokaryotes, over 80% of the whole diversity is related to the domain Bacteria. The commonly identified bacterial phyla include *Proteobacteria, Firmicutes*, and *Bacteroidetes*. The latter phylum has shown hydrolytic activity, and it seems that the members of this phylum dominate under a low level of volatile fatty acids (VFAs), salts, and ammonia at mesophilic temperature in anaerobic digesters [25,26]. The other bacterial phyla such as *Actinobacteria, Planctomycetes, Chloroflexi, Fibrobacteres, Deferribacteres, Fusobacteria, Synergistetes, Nitrospira, Acidobacteria, Tenericutes, Spirochaetes, Verrucomicrobia*, and *Thermotogae* do sometimes exist. Regarding the domain Archaea, most identified phylum in the anaerobic digesters is the phylum *Euryarchaeota*. However, there are some new microorganisms that are not assigned to any microbial taxonomy and introduced as the ‘*Candidatus’* [4,8,13]. Likewise, unculturable ‘*Candidatus’* has been detected in the anaerobic digesters (e.g. OP10, BA024, OP8, TM6, EM3, OP3, and OS-K in the domain Bacteria) [27].

The literature revealed that most studies focused on the optimal conditions for biogas production and waste remediation [28–31]. Such reviews are informative, but none of them comprehensively discusses the microbiological aspects of AD platforms in which new bio-products with various biotechnological applications can be produced. Discussion on digester configurations and operating conditions of anaerobic digesters can be found in elsewhere [9,17,32–34].

This review was aimed to provide an inclusive vision of AD microbiology, the function of microbial communities and various factors involved, novel bio-products, and recent advances of the AD process. This work focused on bio-products generation from the AD process with special emphasis on process microbiology, assessment of microbial communities, and factors affecting their abundance. For this purpose, it was systematically reviewed how microbial communities function and relationships led to various bio-products production during AD. In addition, this review concluded with perspectives and challenges highlighting future research directions.

## 2. Anaerobic digestion for methane production

**Figure 2.**
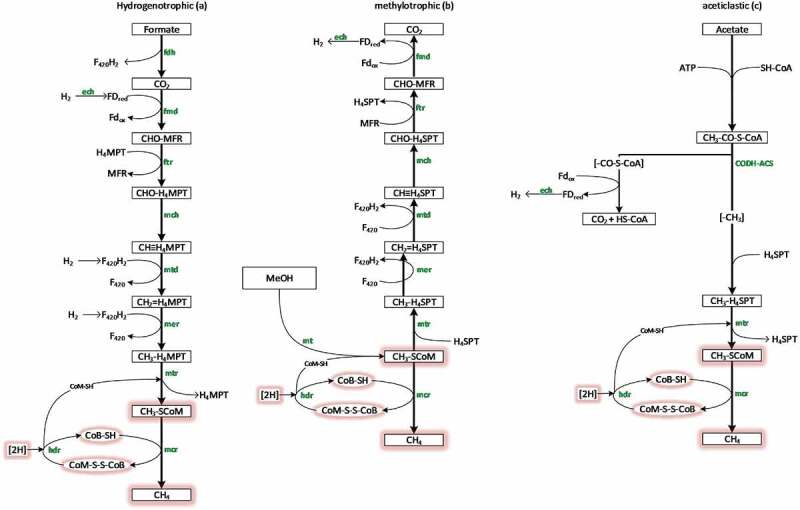
Methanogenesis pathways: Hydrogenotrophic (a), methylotrophic (b), and aceticlastic (c). The methanogenesis common reactions are marked red. *fdh*: formate dehydrogenase; *fmd*: formylmethanofuran dehydrogenase; *ftr*: formylmethanofuran-tetrahydromethanopterin formyl-transferase; *mch*: methenyl-tetrahydromethanopterin cyclohydrolase; *mtd*: methylenetetrahydromethanopterin dehydrogenase; *mer*: methylenetetrahydromethanopterin reductase; *mtr*: methyl-tetrahydromethanopterin S-methyltransferase; *mcr*: methyl-coenzyme M reductase; *mt*: methyltransferase; *hdr*: heterodisulfide reductase; *ech*: energy-converting hydrogenase; CODH-ACS: CO dehydrogenase/acetyl-CoA synthase. The figure was adapted from Niu et al. [303].

One of the final products of the AD process is methane. Understanding the whole AD process and microorganisms involved provide critical information for optimization of industrial-scale of AD system. Methanogenesis originated so early and is carried out via three pathways that depend on coenzyme M (CoM) and methyl–coenzyme M reductase (MCR), a key enzyme in methanogenesis. In Figure 2, methanogenesis pathways were shown in details. These pathways include (1) methylotrophic methanogenesis; (2) acetoclastic methanogenesis; and (3) hydrogenotrophic methanogenesis through CO_2_ reduction [35–38]. The latter pathway is found in most methanogens, but the orders *Methanosarcinales* and *Methanomassiliicoccales* contain some species that are not able to produce methane via CO_2_ reduction. The other two pathways are found in the order *Methanosarcinales*. Moreover, a few members of this order can utilize acetate as a substrate for methane production [39,40]. It seems methylotrophic methanogenesis is not the principal route of methane formation due to possible competition with other microbial communities [41,42]. In general, in this pathway, methane is produced via demethylation of compounds containing methyl group such as methanol, dimethylamine, or monomethylamine [43]. Methylotrophic methanogens are classified into two groups based on the cytochromes. Those methylotrophic methanogens that do not have cytochromes are strictly H_2_-dependent, while the group with cytochromes can oxidize methyl groups to CO_2_ [44].

Through hydrogenotrophic methanogenesis (ancestral pathway of methane formation) [44], up to 28% of methane content is generated in the anaerobic system, while up to 72% of the methane production is driven by the activity of acetoclastic methanogens; however, substrate type may affect these percentages [25]. However, hydrogenotrophic methanogens are more resistant to environmental stresses than acetoclastic ones. For example, in the study by Dong et al. [45], high-throughput sequencing using 16S rRNA analysis showed that hydrogenotrophic methanogenesis continued stably at higher temperature. Moreover, the obtained results revealed that at elevated temperatures, the dominant archaeal community composed of hydrogenotrophic *Methanothermobacter*, and syntrophic bacterial genera changed from *Coprothermobacter* and *Thermodesulfovibrio* at 55°C to *Thermodesulfovibrio* at 70°C.

Generally, archaea are the major microorganisms involved in biomethane production that are very sensitive to various environmental parameters. Therefore, it is of significance to focus on details of the process, archaeal communities, physiological aspects, microbial interactions, process inhibitors and inducers, and other factors [40]. For instance, it seems the dominance of acetoclastic and hydrogenotrophic methanogens is related to the variations and shifts of different VFAs [46].

### 2.1. Microbiology of anaerobic digestion for methane production

In natural environments such as marsh lands, river bottoms, deep lakes, ocean vents, ruminants’ gut, and hot springs, methane production has been going on for millions of years through microbial process. The domain Archaea includes the most important genera that are responsible for methane production in the anaerobic digesters. Generally, the members of this domain grow under extreme habitats but may be sensitive to changes in environmental conditions. Archaea are widespread in various environments from the soil, geothermal systems, and wetlands to animal guts and wastewater treatment plants [39,44]. They are a unique group of microorganisms that are distinguished from bacteria due to different membrane lipids, distinctive ribosomal RNA, and the absence of peptidoglycan in the cell wall. This domain contains 17 phyla: *Aenigmarchaeota, Aigarchaeota, Bathyarchaeota, Crenarchaeota, Diapherotrites, Euryarchaeota, Hadarchaeota, Huberarchaea, Hydrothermarchaeota, Korarchaeota, Lokiarchaeota, Nanoarchaeota, Nanohaloarchaeota, Thaumarchaeota, Thermoplasmatota, Undinarchaeota*, and *Verstraetearchaeota*. Also, three proposed phyla include *Incertae sedis* 374, Ince*r*tae *sedis* 549, and *Incertae sedis* 586 possessing no certain place in the domain Archaea [39,44,47,48].

The metabolism feature of the domain Archaea displays a substantial diversity of chemolithotrophy and chemoorganotrophy that employ fermentation, anaerobic respiration, or aerobic respiration. The capability of archaea for methane production and their high sensitivity to oxygen are both unique characteristics specifying methanogens from other species. Generally, methanogens belong to the phylum *Euryarchaeota* and, the genera include *Methanosarcina, Methanobacterium, Methanocaldococcus*, and *Methanopyrus*. However, methanogens exhibit high diversity in morphology and physiology as classified in seven orders; *Methococcales, Methanomassiliicoccales, Methanomicrobiales, Methanopyrales*, and *Methanosarcinales* [8,11,39]. A brief description of each order is provided in Table 2. Interestingly, the University of Wrocław database provides valuable information for more than 150 methanogenic species that is accessible through (http://phymet2.biotech.uni.wroc.pl/) [49]. Among these orders, *Methanobacteriales, Methanomicrobiales*, and *Methanosarcinales* are the most frequent orders detected in the anaerobic digesters, while *methanococcales* are rarely found. Moreover, in the anaerobic digesters with high organic loading rates and ammonia levels, the members of the order *Methanoplasmatales* were detected, but the other orders of the phylum *Euryarchaeota* were not detected [50–52].

**Table 2.** Some characteristics of methanogenic orders of the domain Archaea

Order	Belong to the Phylum	Type Genus	Type Species	General Phenotypic Characteristics of Type Genus							Reference
				Cell Morphology	Endospore	Motility	Gram Reaction	Optimum Growth Temperatures (°C)	Optimum Growth pH	mol% G + C of the DNA	
*Methanobacteriales*	*Euryarchaeota*	*Methanobacterium*	*M. formicicum*	Curved, crooked, or straight rods, long to filamentous	-	-	+	37–45	ND	32–61	[283]
*Methanocellales*		*Methanocella*	*M. paludicola*	Singly, almost rod-shaped, coccoid cells in late-exponential culture	ND	-	-	35–37	7	56.6	[284]
*Methanococcales*		*Methanococcus*	*M. vannielii*	Irregular cocci	ND	+	Cells lyse	35–40	6–8	29–34	[283]
*Methanomassiliicoccales*	*Thermoplasmatota*	*Methanomassiliicoccus*	*M. luminyensis*	Regular cocci	ND	-	+	37	7.6	59.93	[285]
*Methanomicrobiales*	*Euryarchaeota*	*Methanomicrobium*	*M. mobile*	Singly, in pairs, short, straight to slightly curved, irregular rods	-	±	-	40	6.1–7	48.8	[283]
*Methanopyrales*		*Methanopyrus*	*M. kandleri*	Singly and in chains rods	-	+	+	98	6.5	60	[286]
*Methanosarcinales*		*Methanosarcina*	*M. barkeri*	Irregular spheroid bodies, alone or typically in aggregates of cells, sometimes occur as large cysts with a common outer wall surrounding individual coccoid cells	-	-	±	30–40 for mesophiles 50–55 for thermophiles	ND	36–43	[283]

+, Positive; -, Negative; ±, Variable; ND, not determined.

Due to the high intolerance of methanogens to oxygen, methanogens are obligatory anaerobic microorganisms and, strictly anoxic protocols such as ‘Hungate’ technique should be employed for their isolation, purification, and cultivation in the laboratory [14]. Most of methanogens grow optimally under mesophilic conditions, but some species are extremophiles and they can grow under harsh conditions such as high temperatures, low pH, or high salinity [36,39]. From the phenotypical point of view, methanogens show a wide-range of morphological diversity from rods and cocci to curved rods, spirals, coccoid, sarcina, lancet, cuboid, irregular clusters of cells, aggregates, angular plate, plate, pairs, chains, or filaments with various sizes. For example, in the genus *Methanosarcina*, spherical cells are grouped in a large packet and form a sarcina shape conversely; *Methanosaeta* forms rod-shaped cells [3]. Additionally, they can be motile or non-motile and protect the cells by S-layer [36,47].

Based on the methanogenesis pathways, methanogens are grouped as acetoclastic, methylotrophic, and hydrogenotrophic methanogens. *Methanosaeta* and *Methanosarcina* (acetoclastic methanogens) from the families *Methanosaetaceae* and *Methanosarcinaceae* are extremely vulnerable to process inhibitors such as free ammonia and VFAs [38,53], while hydrogenotrophs belong to the orders *Methanobacteriales, Methanococcales, Methanomicrobiales* and *Methanosarcinales*. Some important examples of hydrogenotrophic methanogens were found in the genera *Methanobrevibacter, Methanocorpusculum, Methanospirillum*, and *Methanobacterium* that can utilize compounds such as formate as the energy source and some alcohols as the electron donors [8,25,35,54,55].

In general, acetoclastic methanogens such as *Methanosarcina* are prevailing archaea in anaerobic digesters, whereas, *Methanothrix soehngenii*, a species of the order *Methanosarcinales*, can degrade acetate in the absence of *Methanosarcina*. However, *Methanosarcina* will be dominant at high concentrations of the substrate in the anaerobic digesters. This genus shows a higher growth rate and contains different cytochromes from other methanogens that enabling it to reduce methylated compounds such as methanol [9,40,56]. Despite the shorter doubling time (1–2 days) of *Methanosarcina* on acetate, *Methanosaeta* has a longer doubling time on acetate (4–9 days). Hence, with a short SRT, it is likely possible that the cells of *Methanosaeta* would washout from the system and therefore, *Methanosarcina* would be dominant in the anaerobic digester. Based on this fact, alteration of various parameters such as SRT, configuration of digester, acetate concentration, or mixing speed in the anaerobic systems favor *Methanosarcina* growth [57]. Generally, when the hydrogenotrophic methanogens are dominated in the anaerobic digesters, it may be concluded that hydrogen or formate is used as the main substrate rather than acetate for methane production [58].

Regarding the high diversity of methanogens, some species utilize various substrates such as formate, hydrogen, acetate, methanol CO_2_, CO, dimethyl sulfide, dimethylamine, 2-propanol and 2-butanol, propanol, butanol, or trimethylamine to produce methane. The most dominant and abundant species in anaerobic digesters are hydrogenotrophic species such as *Methanobacterium formicium* and *Methanobrevibacter arboriphilus* that play an important role in methane production. As a consequence, both types of methanogens are necessary for the effective consumption of hydrogen produced in the previous steps. However, acetoclastic methanogenesis is regarded as the rate-limiting stage during the AD process [12,47].

#### Microbial interactions during methanogenesis

Methanogenesis in the anaerobic digesters is a complicated process that requires coordinated metabolic activities among various microbial communities, even non-methanogenic microorganisms. For an enhanced understanding, it is necessary to focus on the noteworthy interactions occurring among bacterial and archaeal communities during methanogenesis. Moreover, the whole microbiota of an anaerobic digester is not only essential for various phases of the AD process but also stabilizes the redox potential (E_h_) of the system. The importance of redox potential becomes apparent when the system requires stabilizing methanogens for methanogenesis. For example, some especial fastidious methanogens can only grow in an environment with the E_h_ of −300 mV. However, environmental parameters and nutritional sources for microbial growth play an important role in influencing methanogenesis efficiency [40].

#### Granule formation

Granule formation is one of the important microbial interactions in the anaerobic digesters especially, in the up-flow anaerobic sludge blanket (UASB) reactors. The microbial granule resembles a filamentous consortium through which fluids and gases can flow slowly. This strategy helps microbial cells to protect themselves in the stressful environments and from technical point of view; stable granules can increase anaerobic digesters efficiency. Based on the digester types and reactor loading rates, mechanisms of granule formation may differ from each other. However, these mechanisms are not fully understood, and the investigations are still in their infancy stage. Nevertheless, it was shown that the addition of some ions with proper amounts (not high concentration of metals) may improve the rate of granulation. These ions include ferrous (12–84 mg/L), calcium (80–200 mg/L), magnesium (12–120 mg/L), and aluminum (300 mg/L) [59]. Another proposed mechanism is related to cell-to-cell signaling. It is supposed that one of the granule microorganisms, perhaps propionate-oxidizing bacteria, activates aggregation [60].

In general, a granule consists of three layers: (1) a packed outer layer of the methanogenic cells such as *Methanococcus* and *Methanosarcina*; (2) a central layer of methanogenic ovoid cells with intercellular spaces; and (3) internal cavities with non-methanogenic microorganisms. One of the important archaeal genera is *Methanosaeta* that significantly aids in the texture formation of granules. The members of this genus form filamentous cells and exhibit a higher affinity to acetate that can exclusively consume it in comparison to *Methanosarcina* [12]. Furthermore, in a study, the microbial community of UASB granules was investigated by 16S rDNA clone library, real-time quantitative-polymerase chain reaction (RTQ-PCR), and RFLP (restriction fragment length polymorphism). The obtained results revealed *Methanosaeta* and *Methanobacteria* were dominant archaea in the granules. However, Gram-positive bacteria with low G + C content and ε-*Proteobacteria* were also detected in the granules [61]. Other microorganisms may be present in the granules. For example, the presence of bacteriophages can disintegrate the granules, while protozoa may play a role in the controlling of bacterial cell numbers [59].

Micro-spatial structures that are created via granules and extracellular polymeric substances (EPS) provide niches for various microbial communities and maintain the function of the communities. These structures are important for metabolite exchange among microorganisms living in the granules [13,59]. Many factors may affect the granules formation and function. For example, an increase in the mixing and high shear may reduce granules development. Furthermore, it is reported that the high levels of mixing changed the dominance from *Methanosaeta concilii* to *Methanosarcina* spp. [62].

#### Syntrophic relationship between acetate-forming bacteria (acetogens) and methanogens

A considerable syntrophic relationship occurs between methanogens and acetate-forming bacteria because the latter provide the main substrates (acetate and hydrogen) for methane production. Acetogens which belong to various bacterial genera can convert products obtained from acidogenic phase into acetic acid, CO_2_, and H_2_ [63,64]. Acetogens are strictly anaerobes that are considered quite susceptible to the changes in the process environment compared to other bacteria [13,35]. Metabolism in the acetogens can be heterotrophic or autotrophic. Autotrophic acetogens can utilize CO_2_ and sometimes CO as the carbon source for cellular synthesis. Conversely, heterotrophic acetogens can use organic compounds such as formate or methanol as the sole carbon source. Many heterotrophic acetogens are isolated from sewage sludge that mostly belonged to the genera *Clostridium* and *Acetobacterium* [8]. Remarkably, acetogens are diverse but commonly belong to the low G + C branch of the phylum *Firmicutes* including the genera *Aminobacterium, Aminomonas, Pelotomaculum, Syntrophobotulus, Syntrophomonas*, S*yntrophospora, Syntrophothermus, Thermoacetogenium, Carboxydocella, Thermosinus, Thermosyntropha*, and *Moorella* [4]. Furthermore, the phylum *Proteobacteria* contain syntrophic members belong to the genera *Pelobacter, Smithella, Syntrophobacter, Syntrophorhabdus*, and *Syntrophus* [8].

Acetogens can be divided into two groups: (1) those that produce hydrogen and acetate from organic acids and carbohydrates (e.g. *Anaerovorax odorimutans, Macellibacteroides fermentans, Saccharofermentans acetigenes, Proteiniphilum acetatigenes, Levilinea saccharolytica, Hydrogenispora ethanolica*, and *Hydrogenophaga carboriunda*) and (2) those that consume hydrogen and CO_2_ to produce acetate such as *Acetobacterium wieringae, Acetobacterium woodii, Acetogenium kivui, Clostridium aceticum, Clostridium thermoautotrophicum*, and *Sulfurovum riftiae* [25]. The formation of acetic acid by acetogens from group one produces a large amount of hydrogen that decreases the pH of the reactor. This hydrogen can be consumed in two ways: (1) during the formation of methane and (2) during the formation of organic acids such as butyric and propionic acids. Butyrate and propionate are two VFAs for acetate production; however, conversion of these VFAs to acetate cannot take place spontaneously due to positive Gibbs free energy of the reaction. In the anaerobic digesters, propionate can be fermented by *Syntrophobacter wolinii* to acetate. The cells are gram-negative rods, motile and strictly anaerobic that can survive in anaerobic digesters; however, if propionate is not fermented by these types of bacteria, it will accumulate in the cells. The propionate accumulation is an indicator of stress in the anaerobic systems. The same as propionate, butyrate accumulation indicates a stressful situation in the anaerobic digesters [4,65]. In this regard, syntrophic relationship of propionate-degrading bacteria and methanogens together has an excessive impact on the stability of the system. It is found that these two groups of microorganisms are in close association [60]. For example, 66,reported a syntrophic relationship between *Syntrophobacter* and *Methanobrevibacter* [59]. Commonly, in an anaerobic system, VFAs are produced during AD process; however, high concentration of these acids may disturb the system. The continuous accumulation of VFAs decreases the pH and results in souring and eventually failure of the system. Among VFAs, the effect of propionic acid on system disruption is stronger than other VFAs. Based on the feedstock, initial inoculum, operating conditions, and reactor configuration, concentration of VFAs may vary. For example, maximum inhibitory concentrations of propionic acid fluctuate from 0.8 to 21.6 g/L [67,68].

Ordinarily, the acetogenesis process is considered thermodynamically unfavorable if the hydrogen partial pressure rises above 10^–3^ atm. Therefore, it is required to keep the partial pressure of hydrogen below this threshold. In the anaerobic digesters, hydrogen removal takes place efficiently by hydrogenotrophic methanogens, sulfate-reducing Bacteria (SRB) and homoacetogens [12]. Comprehensively, interspecies hydrogen transfer is a critical factor in the syntrophic relationship that can prevent the hydrogen build-up as it is consumed by methanogens to reduce carbon dioxide and, in general, keep the anaerobic system in balance. Moreover, it helps acetogens grow because they can survive at very low concentrations of hydrogen [8,36]. In addition to interspecies hydrogen transfer, interspecies formate transfer has been reported in the syntrophic relationship of acetogens and methanogens. These mechanisms are known as indirect or mediated interspecies electron transfer (MIET) [69,70]. Moreover, direct interspecies electron transfer (DIET) through bacterial pili, cytochromes, or nanowires (cell–cell contact) is possible among syntrophic microbial communities [11,42,71]. However, various mechanisms of electron transfer may co-exist in the same microbial cells [72].

#### MIET in anaerobic digestion process

##### Interspecies hydrogen transfer

As hydrogen concentration is important for both acetogens and methanogens, its transfer is a usual phenomenon occurring in syntrophic interactions. As well, the metabolite exchange helps the system to have a balanced function [60,71,73]. Interspecies hydrogen transfer takes place between two cells that are not capable to oxidize organic material individually. Therefore, they exchange electrons via hydrogen to degrade organic materials [73]. Hydrogen is a small molecule that diffuses easily, but its solubility is low; so, the transfer distance for hydrogen is fairly low (10 μm). However, this distance has a significant role in the selection of electron carriers by microorganisms. If microbial cells have a distance less than 10 μm, they use hydrogen for the electron transfer. This point reveals that the close association of acetogens and methanogens in the anaerobic granules, soils, or anaerobic aquatic systems is preferred and, therefore, hydrogen is the most common electron carrier [71,74]. However, hydrogen concentration can affect electron transfer through hydrogen [72]. Renslow et al. [75] demonstrated that the hydrogen transfer rate depended on its diffusion flux, while electron carrier flux in DIET relied on the conductive pili or conductive materials.

##### Interspecies formate transfer

In a syntrophic relationship, both hydrogen and formate can be produced by microbial communities. Interspecies formate transfer can be favoured when the distance of microbial cells is high. This mechanism of electron transfer plays an important role in the syntrophic propionate-degrading co-cultures. Based on the finding, when the microbial cells take a distance of more than 10 μm, formate will be used as an electron carrier [60,74]. Also, electron transfer via formate is an advantageous mechanism during syntrophic oxidation of fatty acids in floc system. In general, interspecies formate transfer rate is higher than interspecies hydrogen transfer rate based on the higher diffusion of formate [72].

##### DIET in anaerobic digestion process

The importance of DIET was determined in 2010 by Summers et al. [73]. To investigate DIET, they deleted the *hyb* gene which encoded uptake hydrogenase protein in *Geobacter sulfurreducens* that made this strain unable to consume hydrogen. The mutant strain of this species was co-cultured with *Geobacter metallireducens* to metabolize ethanol. The cell aggregate was formed during 21 days in comparison to the 7 months needed for the co-culture of wild-type *G. sulfurreducens* and *G. metallireducens*. The obtained results revealed that an alternative electron transfer occurred in the mutant co-culture when an interspecies hydrogen transfer was not possible. Moreover, it was shown that the deletion of the *pilR* gene in *G. sulfurreducens* had a sufficient effect on the stimulation of aggregate formation. The *pilR* deletion enhanced the *omcS* gene expression, which encodes a multiheme c-type cytochrome associated with conductive pili that stimulate electron transfer to insoluble iron (III) oxide. Based on these results, a possible model for electron exchange between the cells of *G. sulfurreducens* and *G. metallireducens* was proposed as OmcS of *G. sulfurreducens* accepted electrons from outer-surface c-type cytochromes of *G. metallireducens* directly. In general, DIET is a phenomenon in which electrons transfer takes place from one cell to another cell without mediating of any reduced molecules such as hydrogen or formate. The electron transfer between two species strongly depends on the durability of the cell contact. The occurrence of DIET could explain the syntrophic relationship of the microbial cells in the anaerobic aggregates. Moreover, DIET has some distinctive advantages such as higher speed of electron transfer and no requirement for hydrogen/formate shuttles. Also, by increasing hydrogen partial pressure, DIET is the most likely mechanism for electron transfer. However, in the suspended anaerobic microbial consortia, interspecies hydrogen or formate transfer may favor if they are not limited by a severe prerequisite for inter-microbial distances [69,71,72]. The electron transfer rate of DIET depends on interspecies distance, amount of cytochromes and nanowires, microbial community resistivity, and cell-nanowire cofactor electron transfer rate constant [72].

With the discovery of importance of DIET, researchers tried to enhance this mechanism of electron transfer to improve methane production, shorten the start-up time, prevent system souring, and stabilize the whole system during the AD process [69,76]. For example, adding conductive materials such as graphene, granular activated carbon (GAC), biochar, zero-valent metals, or hematite to the anaerobic digesters can promote DIET among methanogens (mostly *Methanosaeta, Methanosarcina, Methanoregula, Methanobacterium, Methanospirillum*, and *Methanolinea*) and exoelectrogenic bacteria such as *Geobacter, Smithella, Thauera*, and *Syntrophomonas*. In addition, some species of *Bacteroides* and *Streptococcus* may participate in DIET. Also, putative e-pilin (electrically conductive pilin) genes were detected in *Desulfobacterium, Deferribacter, Geoalkalibacter, Desulfobacula*, and *Syntrophus* that making them probable partners for taking part in DIET [69,71,72,77–80]. There is need for further research on monitoring of DIET in the anaerobic systems. Lately, molecular techniques such as transcriptomics, analysis of microbial community structure by 16S rDNA sequencing, or genomic analysis, high-resolution imaging approaches, the characterization of spatial distribution, cellular, and electrical properties, change of conductivity, and performance of the system aid us to gain more insight on the occurrence of the DIET among the anaerobic microbial communities [76]. Transcriptomics is the most direct method for DIET evaluation [72,81]; however further research on a combination of various techniques needs to carry out to gain insight about DIET in the microbial communities of the anaerobic digesters.

##### Sulfate-reducing bacteria (SRB): importance in the anaerobic digestion process

Among anaerobic microorganisms living in the anaerobic digesters, SRB play a significant role that may result in the inhibition of methanogenesis. This group of microorganisms competes with methanogens to achieve hydrogen and acetate, the same substrates for methanogens, to reduce sulphate [47,57]. Thoroughly, two major types of SRB can be demonstrated: (1) those can oxidize the substrates to acetate like the genera *Desulfobulbus, Desulfomonas, Desulfotomaculum*, and *Desulfovibrio* and (2) those which can oxidize organic acids, including acetate to CO_2_. This type of SRB comprises *Desulfobacterium, Desulfobacter, Desulfosarcina, Desulfococcus*, and *Desulfonema*. SRB can simply obtain the substrates compared to methanogens; therefore, they can quickly be the dominant community in the anaerobic digesters. However, SRB may make syntrophic association with hydrogenotrophic methanogens to degrade propionate or butyrate. In addition, SRB can work independently in the anaerobic digesters and compete with methanogens [25]. One of the strictly anaerobic genera of SRB is *Desulfotomaculum* that can reduce sulphate to hydrogen sulphide (H_2_S). This toxic and corrosive gas has an inhibitory effect on the growth of methanogens and acetogens and may reduce the rate of methane production and produce malodor in the reactor [12,47]. By microaeration, sulphide/sulphur-oxidizing bacteria (SOB) such as *Acinetobacter, Halothiobacillus, Sulfuricurvum*, or *Thiobacillus* can be predominant genera that their activities result in the H_2_S removal from the anaerobic digesters. But the oxidation of H_2_S to elemental sulphur or other products is associated with pipe clogging and a low rate of methanogenesis that are practical challenges during microaeration of the system [82]. In addition to SRB, iron-reducing and nitrate-reducing bacteria can compete with acetoclastic methanogens. For this purpose, iron and nitrate should be present to accept electrons. *Deferribacter* and *Denitrovibrio* are common genera of iron-reducing and nitrate-reducing bacteria, respectively [4,83].

##### Phages and their effects on the microbial communities of anaerobic digestion

The phages that infected bacterial cells are abundant in natural and human-made environments and can affect the structure, abundance, and dynamics of microbial communities. Anaerobic digesters are no exception, and phages may be present in these systems. In addition to bacteriophages, archaeal viruses (archeoviruses, infect archaeal cells) may be found. To the best of our knowledge, no report was detected about mycoviruses (infect fungal cells) in the anaerobic digesters. Overall, the details about these viruses occurrence in the anaerobic digesters are limited, most likely since detection, purification, and characterization of the phages need advanced equipment and methods such as fluorescence assay, transmission electron microscopy (TEM), and field inversion gel electrophoresis (FIGE). However, understanding the phages ecology and function are important as they can simplify genetic exchanges among microbial communities and influence metabolic diversity among them [84–86].

Various bacteriophages and archaeal viruses are reported to infect microorganisms associated with AD. *Desulfovibrio* and *Clostridium* can be infected by myophages and siphophages, respectively, [84]. Among methanogens, it is known that *Methanosarcinales* do not harbour phages, while *Methanococcus* and *Methanobacterium* can be infected by siphophages [84,85]. In a study, 87,reported dominant viral families during AD to be *Podoviridae* (16.2%), *Myoviridae* (36.0%), and *Siphoviridae* (42.4%), where the first family can only infect bacterial hosts. In Table 3, some examples of methanogens infected by archaeal viruses are presented. It can be concluded that the viral outbreak of infected methanogens is the probable reason for the instability of microbial communities and the sudden breakdown of the digesters. It could be one the reasons that anaerobic digesters lose their functionality without any recognized reason. However, a lower load of these viruses may not significantly affect digester performance [84,88,89]. It is assumed that these viruses target the most rapid growing microbial communities in the anaerobic digesters (‘kill the winner’ strategy) and stimulate microbial diversity. However, there is an information gap about viral infection mechanisms and the factors that affect these mechanisms [90,91].

**Table 3.** Some examples of methanogens infected by archaeal viruses belong to the order *Caudovirales.*

Methanogen	Virus Name	Reference
*Methanobacterium thermoautotrophicum* Marburg	psiM1	[287]
*Methanothermobacter wolfeii*	psiM100	[288]
*Methanobacterium* species	phiF1 and phiF3	[289]
Several *Methanobacterium* species	phiF1	

One of the newest omics approaches that provide valuable details is viromics, in which virome analysis is performed. By comparison of the viromes to clustered regularly interspaced, short palindromic repeats (CRISPRs), likely hosts for bacteriophage and archaeal viruses can be determined [85,92]. Moreover, by detecting viral communities and their relationship with methanogens, an improvement in the stability of anaerobic digesters may happen. In addition, performing a phage-based treatment can regulate the frequency of target microbial groups that resolve system problems such as bulking and foaming, or operate the system toward an expected product [93–96].

### 2.2. Assessment and identification of methane-producing microbial communities

In general, assessment of a special microbial community in the anaerobic digesters is a not simple task, as some microbial cells attach to surfaces, and some create consortia with different properties in comparison to single cells. Therefore, for microbial analysis in the anaerobic digesters, it is required to extract and isolate each microbial group. However, for viable biomass, assessments are designed based on the common constituents of the cell. For example, living cells can be measured by a luciferin-luciferase or fluorescence assay that estimates whole viable biomass in the anaerobic digesters. Another technique for the assessment of microbial biomass is the analysis of signature lipids, which can differentiate prokaryotic from eukaryotic microorganisms and estimate the ratios of aerobic and anaerobic microorganisms in the anaerobic system. Moreover, the metabolites produced in each stage in AD can be measured by analytical methods such as high performance liquid chromatography (HPLC) and gas chromatography (GC) that makes it possible to monitor each stage of the AD process [40].

It is known that methanogenic microorganisms belong to the domain Archaea that are quite difficult to be cultivated and isolated under laboratory conditions due to their requirement to the low redox conditions. Providing low redox conditions can be performed by removing or replacing oxygen from the growth medium. In the last decades, various strategies such as co-cultivation or six-well plate system were developed to isolate strict anaerobic methanogens such as *Methanomassiliicoccus*, but these methods could not be enough for identification of whole system microbiota [97,98]. Thus, molecular methods and techniques are promising way for identification of this group of microorganisms. The breakthrough development of high-throughput sequencing technologies has facilitated the identification of microorganisms. Obviously, microbial communities in the anaerobic digesters are diverse and abundant, but conventional methods (culture-dependent) cannot detect the majority of species existing in the anaerobic digesters. Additionally, the pure culture of microorganisms involved in the AD process cannot reveal the competition, synergism, or interactions that occur among microbial communities. Conversely, culture-independent methods that are less laborious and more rapid provide more details and data about microbial structure, diversity, dynamics, functioning, and quantification. Moreover, uncultivable microorganisms can be detected by this type of molecular methods. For this purpose, the analyses of bacterial and archaeal communities based on the generation of 16S rRNA gene clone libraries and Sanger sequencing of 16S rDNA amplicons have been the most common methods applied recently [8,15,99,100].

The rDNA-based molecular methods like T-RFLP (terminal-RFLP) allow rapid fingerprinting of archaeal populations [101] but have not been fully successful for methanogenic and non-methanogenic lineages [102,103]. The structure of a microbial community is well defined by fingerprinting techniques, such as denaturing gradient gel electrophoresis (DGGE), stable isotope probing (SIP), quantitative real-time PCR (qPCR), temperature gradient gel electrophoresis (TGGE), T-RFLP, ribosomal intergenic spacer analysis (RISA), and DNA microarrays that are important to find out about the functional properties of a microbial community in the AD process [13]. Molecular techniques facilitate better characterization and understanding of prevalent species in an anaerobic microbial community, their metabolic capacity, and their interspecies interactions, which can lead to a better control of microbial-based production in such systems [6,8].

In anaerobic processes, methane is produced by methanogenic archaea and methane oxidizing archaea (MOA) groups. This process is expressed by methyl coenzyme M reductase (MCR) consisting of *mcrA* operon. This operon has composed of two alpha (*mcr*A), beta (*mcr*B), and gamma (*mcr*G) subunits [6,104]. The genes of both *mcr*A and *mrt*A, which is isoenzyme methyl-coenzyme M reductase, are highly conserved [105,106]. The *mcr*A gene has been frequently screened to identify the methanogenic organisms [104,107–110]. Table 4 presents various molecular-based methods for identification of methanogens. Convincingly, there is a substantial complexity among microbial communities of the AD that requires more research to realize the exact function at the species level.

**Table 4.** Molecular-based methods for identification of methanogens

Method	Target Sequence	Identified Methanogens	Reference
PCR	16S rRNA	*Methanobrevibacter* sp., *Methanosaeta concilii* *Methanolinea tarda* *Aciduliprofundum boonei*	[290]
	16S rRNA, with using primers 27 f and 1492 r	Archea; *Methanobacterium palustre* *Methanobacterium aarhusense* Bacteria; *Methylocystis* *Desulfovibrio putealis* *Petrimonas sulfuriphila* *Ottowia thiooxydans*	[291]
T-RFLP analysis	rRNA-encoding genes (rDNA)	*Methanosarcinaceae* *Methanosaetaceae*	[102]
	rDNA amplicons	*Methanosarcinaceae* *Methanosaetaceae* *Methanomicrobiaceae* *Methanobacteriaceae*	[103]
	*mcr*A and *mrt*A sequences	*Methanobacterium bryantii* *Methanosarcina* spp. *Methanosaeta concilii* *Methanospirillum hungatei* *Methanoculleus thermophilus*	[108]
PCR	*mcr*A	*Methanobacteriales* *Methanomicrobiales* *Methanosarcinales* Unclassified euryarchaeota	[292]
RFLP	*mcr*A and 16S small subunit rRNA gene sequences	*Methanobacterium formicicum* *Methanothermobacter thermoautotrophicus* *Methanobrevibacter arboriphilicus* *Methanoculleus bourgensis* *Methanospirillum hungatei* *Methanocorpusculum bavaricum* *Methanocorpusculum parvum* *Methanomicrobium mobile* *Methanosarcina barkeri* *Methanosaeta concilii*	[293]
PCR	Small subunit rRNA and *mcr*A gene amplification	*Methanothermobacter thermoautotrophicus* *Methanosarcina barkeri* *Methanocaldococcus jannaschii* *Methanospirillum hungatei*	[104]
	*mcr*A and 16S rRNA gene-specific primers	*Methanoculleus marisnigri* *Methanoculleus chikugoensis* *Methanospirillum hungatei* *Methanosaeta concilii* *Methanosaeta thermophila* Uncultured Archaeal	[110]

### 2.3. Factors and inhibitors affecting methanogenic communities

In the AD process, the methane-forming microorganisms diverge extensively in terms of nutritional requirements and irritability to environmental conditions [111]. Failure to sustain the optimum condition for the microorganisms is the fundamental reason of reactor instability in methane production [57]. Due to the low-rate growth of methanogens, it is a perquisite to increase retention time (e.g. more than 12 days) to ensure the establishment of methanogen communities in the anaerobic digesters [47]. However, low feedstocks’ digestibility in the hydrolysis phase of the AD process by hydrolytic bacteria may become rate-limiting step. In this case, it is necessary to pretreat wastes before entering AD. Thermal and mechanical pretreatment, microaeration, adding extracellular enzymes such as cellulase or aerobic bioprocessing are examples of pretreatments that are examined to accelerate the hydrolysis phase of the AD process [4,33,82,112–115].

The increased OLR regularly results in the accumulation of VFAs. High levels of VFAs results in an interruption of biogas production due to the high acidity of the digester. This state clearly shows the shifts toward the bacterial community, particularly *Chloroflexi*. However, this phylum mostly dominates in the municipal wastewater plants, while in the digesters with manure as feedstock, the phylum *Firmicutes* was reported to be the dominant representative [56]. The high levels of VFAs act as inhibitors that decrease the populations of the hydrogenotrophic genera such as *Methanoculleus* and *Methanothermobacter* and increase acetoclastic *Methanosarcina* spp. [8]. In general, inhibitory factors that have a dominant effect on the reactor upset consist of substances such as ammonia, sulfide, heavy metals, as well as environmental factors like, temperature, pH, and concentration [116].

#### 2.3.1. Ammonia

The optimal concentration of ammonia guarantees efficient methanogens activity and increases the stability of AD [117]. Proteins are nitrogenous constituents in feedstock that are degraded to ammonia through digestion processes [118]. A low concentration of total ammoniacal nitrogen (TAN) is indispensable for synthesizing amino acids and nucleic acids, and is eventually vital for microbial growth. Moreover, ammonia acts as the base to neutralize the organic acids provided by the fermentative bacteria, therefore assists in buffering capacity and keeping pH in the neutral condition, which is critical for cell growth [119,120]. However, a high concentration of ammonia (more than 1.7 g/L) inhibits methanogenesis [11,119,121]. The level of pH in AD is an important factor. The optimal pH range in the digestion is 6.5–7.5 to obtain maximum methane yield. However, this optimum level is dependent on the substrate and digestion technique [122,123]. There are various theories for the ammonia inhibition mechanism, such as a shift in cell pH, accession in energy demand for maintenance, and blockage of enzyme reaction [124]. Among the four types of anaerobic microorganisms, methanogens are the most susceptible microorganisms to inhibitory effect of ammonia [118]. Some studies indicate that high ammonia concentration has a more potent inhibitory effect for the acetoclastic methanogens than for the hydrogenotrophic methanogens [125,126]. Moreover, the environmental factors such as substrate concentration, pH, and temperature could inhibit the methanogens and synergize the inhibition effect of ammonia [127–129].

Increasing the total ammonia nitrogen concentration to the range of 1.7–14 g/L is reported to decrease methane yield to half of the optimum value [130,131]. The pH induces the microorganism’s growth plus compound of total ammonia nitrogen [132]. The total ammoniacal nitrogen is most likely converted to free ammonia (FA) in higher pH, and this form is the actual toxic agent [133]. Temperature variation could influence both microbial growth and free ammonia concentration. The high free ammonia concentration represses methanogens more efficiently in thermophilic temperatures than in mesophilic temperatures [134]. Some specific ions such as Ca^2+^, Na^+^, and Mg^2+^ were determined to be antagonist to the ammonia inhibition effect, an incident wherein the presence of ions barricaded the ammonia effect. It was shown that the methane generation from acetic acid was reduced by 20% because of 0.15 M ammonia in the system, whereas a surplus of 0.05 M Na^+^ produced 5% more methane [135,136].

#### 2.3.2. Sulfide

SRB convert sulfate to sulfide in anaerobic digesters [137]. Sulfide can inhibit methanogenesis through two mechanisms. Initial inhibition is owing to SRB rivalry for common organic and inorganic substrates that quench methane generation [138]. Subsequent inhibition is due to the toxicity of sulfide to diverse microbial communities that play a critical role in the methanogenesis [139].

#### 2.3.3. Light metal ions (Na, K, Mg, Al)

Light metal ions exist in the influent of anaerobic digesters. They could be discharged by degradation of organic matters or supplemented as pH modification chemicals [140]. Extreme values diminish the microbial growth and make a negative osmotic pressure for methanogens, which dehydrate the cells and lead to death [141,142].

#### 2.3.4. Heavy metals

A distinctive characteristic of heavy metals is that they are not biodegradable, unlike other toxic elements, and can accumulate to possibly inhibiting concentrations. The inhibiting effect of heavy metals such as cobalt, copper, zinc, cadmium, and nickel is associated with the interruption of enzyme operation and structure. They can interlock with thiol and other parts of proteins that result in cellular malfunction or cell death [141,143].

#### 2.3.5. Antibiotics

The ubiquity of antibiotics and their residues in anaerobic reactors restrain the microbial community and performance. Their inhibiting effect is diverse due to their various action mechanisms and concentrations [144]. Macrolides principally involve roxithromycin, erythromycin, and tylosin. Erythromycin most likely hinders acetate utilization by acetoclastic methanogens; thus, the concentration of acetate increases, followed by subsequent pH drop, resulting in methanogenesis suppression [145]. Roxithromycin seems to restrain more methanogens than hydrolytic bacteria, followed by VFAs accumulation and digestion failure [146].

Tetracyclines including terramycin, aureomycin, and tetracycline do not inhibit methanogens below 25 mg/L, but suppress methane production in concentrations above 500 mg/L [147]. Commonly, antibiotics’ inhibition effect leads to VFAs accumulation in the system, though the presence of different types of VFAs depends on type and concentration. The inhibition effect could be diversified extensively concerning the origin, composition, environmental factors, and conditions of the substrates. Accumulation and synergy of these elements could lead to fermentation failure, as designated by methanogenesis decrement.

## 3. Anaerobic digestion for VFAs production

Nowadays, the recovery of value-added products from wastes is an attractive issue for researchers and engineers. In this regard, VFAs or short carboxylic acids are considered noteworthy products that can be recovered from anaerobic digesters and used in the pharmaceutical, food, textile, and chemical industries. Moreover, they are suitable substitutes for biodegradable polymers production, biofuels production (butanol, ethanol, and biodiesels), and nitrogen removal from wastewater to replace petrochemicals. These acids include C_2_ to C_6_ carboxylic acids including acetic, propionic, butyric, isobutyric, valeric, isovaleric and caproic acids [11,24,148,149]. During the AD process, hydrolysis and fermentation of wastes by facultative anaerobic bacteria result in the production of a wide variety of organic compounds such as organic fatty acids, alcohols, indole, skatole, hydrogen, etc. [24,37,41,150]. This variety is strongly dependent on bacterial species. Thus, any changes in the operation of the system may change bacterial community, and eventually the products. The acidogenic reactions using carbohydrates as substrate can follow different pathways, such as Embden–Meyerhof–Parnas or Entner–Doudoroff, in which pyruvate acts as an electron acceptor for re-oxidation of NADH [8,11,55]. For amino acids’ fermentation, there are two pathways: '(1) Stickland reaction between a pair of amino acids and (2) deamination of single amino acid. In addition, glycerol is the main product of lipid hydrolysis that can be fermented to various fatty acids, alcohols, hydrogen, and CO_2_. The long-fatty acids may degrade via β-oxidation pathway [4,8].

The bacterial community can produce VFAs via several metabolic pathways including acetate-ethanol type, butyrate-type, propionate-type, and mixed acid fermentation that in all pathways, pyruvate is the critical point resulting in different products [151]. From techno-economical point of view, biological production of VFA must be sufficiently affordable with high yield and productivity to compete with petrochemicals [8,47]. In the routine anaerobic digesters, acidogenic reactions take place faster than methanogenesis; therefore, with the high OLR and short HRT, fast-growing microbial community dominates and accumulation of VFAs occurs significantly. Moreover, a range of redox potential between −100 and −200 mV is suggested to optimize VFAs production for higher yield, but at the higher redox potential, propionic acid will be dominant [82]. Another configuration for VFAs production under anaerobic conditions is called dark fermentation (DF) process, which is similar to AD; however, methanogenesis does not occur in this configuration [2,152]. However, for the sustainable production of VFAs, final products should be extracted by various approaches like membrane technology from the system to decrease the product inhibition effect. In practice, the pH adjustment (out of methanogens tolerance) and the usage of methanogen inhibitors are other strategies that favor the higher production of VFAs, but it should be considered that maintaining alkaline conditions in the anaerobic systems may raise operating cost [149,153–155]. Furthermore, the microaeration strategy can be employed for VFAs production instead of methane, hydrogen, and CO_2_ in the anaerobic digesters. In this configuration, obligatory anaerobic methanogens are excluded, and the environment is suitable for facultative anaerobic bacteria producing VFAs [82].

### 3.1. Microbiology of VFAs production

In recent decades, biotechnology, metabolic engineering, and system biology had a great impact on the development of engineered microbial strains for VFAs production from renewable sources. The VFAs can be produced aerobically and anaerobically, but during anaerobic fermentation, higher productivity and yield can be achieved due to less carbon substrate usage for energy generation and microbial cell growth. The most significant VFAs in the anaerobic digesters are acetic and propionic acids, which are required precursors for methane production but formic and butyric acids can also convert to methane [8,12,40]. Acidogenic bacteria can either be aerobes, facultative anaerobes, or strict anaerobes. They include members of enteric bacteria such as *Klebsiella, Citrobacter, Enterobacter*, and *Escherichia, Bacteroidia, Bifidobacteria, Clostridia, Bacilli*, and *Lactobacilli* [10,156–158].

#### 3.1.1. Acetic acid

**Figure 3.**
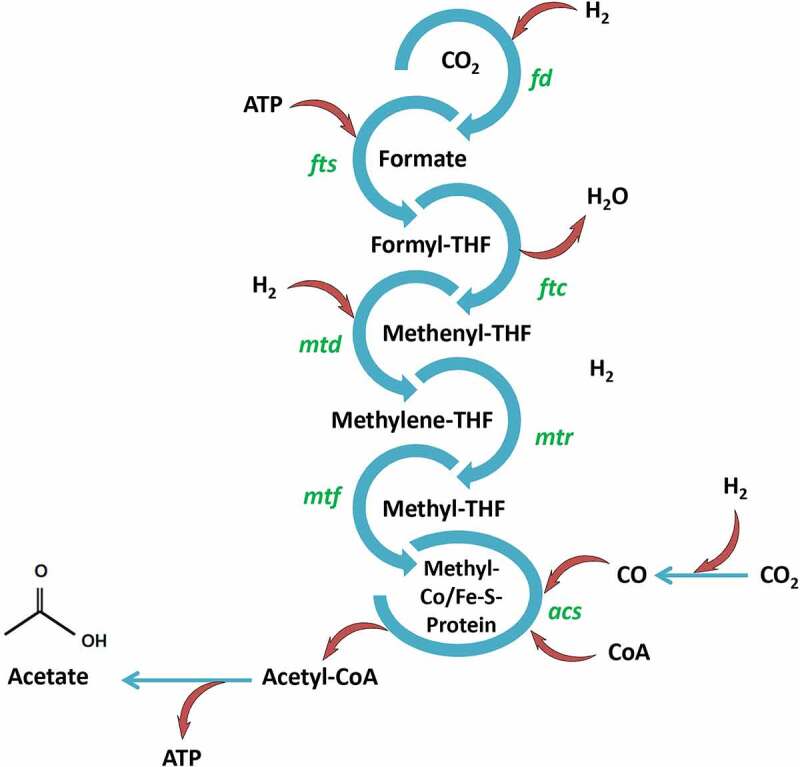
Wood–Ljungdahl pathway. The genes encoding critical enzymes are: *fd* formate dehydrogenase, *fts* formyl-THF synthase, *ftc* formyl-THF cyclohydrolase, *mtd* methylene-THF dehydrogenase, *mtr* methylene-THF reductase, *mtf* methyltransferase, and *acs* acetyl-CoA synthase. THF, tetrahydrofolate.

Under anaerobic conditions, acetogens such as *Moorella thermoacetica, Clostridium formicaceticum, Clostridium aceticum, Acetobacterium woodii*, and *Thermoanaerobacter kivui* can produce acetic acid as the only fermentation product from a variety of hexoses and pentoses. Other important microorganisms responsible for the production of acetic acid are *Streptococcus lactis, Clostridium thermoaceticum, Acetobacter pasteurianus, Acetobacter aceti, Acetobacterium wieringae, Acetomicrobium* and *Gluconobacter* strains [159–164]. Furthermore, the Wood–Ljungdahl pathway (Figure 3) used by anaerobic acetogens is a sustainable method that contributes to the reduction of greenhouse gases (GHGs) and lowering the costs of downstream processing [165,166].

#### 3.1.2. Propionic acid

Propionic acid is usually produced along with acetic and succinic acids in the anaerobic digesters. The microbial community that is responsible for propionic acid production includes propionibacteria. This group of bacteria belongs to the phylum *Actinobacteria* that their members are gram-positive anaerobic rod-shaped cells. The most significant species of propionibacteria are *Propionibacterium freudenreichii* and *Propionibacterium shermanii* which can produce acetic acid by using phosphotransacetylase (PTA), acetate kinase (ACK), acetate-CoA ligase, or acetyl-CoA synthetase. In the obligatory anaerobic *Clostridium propionicum*, propionic acid is produced through the acrylic acid pathway. Other involved species of the genus *Propionibacterium* for propionic acid production are *P. acidipropionici, P. thoenii*, and *P. jensenii* [8,167–169].

#### 3.1.3. Butyric acid

Another beneficial VFA usually reported in the anaerobic digesters is butyric acid (four-carbon), which is widely applied in various industries. Butyric acid can be produced by a wide variety of anaerobic bacterial genera such as *Butyribacterium, Butyrivibrio, Clostridium* (especially *Clostridium tyrobutyricum* and *Clostridium butyricum), Coprococcus, Eubacterium, Fusobacterium, Megasphaera, Roseburia*, and *Sarcina*. In butyric acid fermentation, acetyl-CoA derived from a hexose can be converted to acetate or either butyryl-CoA. The latter is further converted to butyrate by two key enzymes including phosphotransbutyrylase (PTB) and butyrate kinase (BUK) that are frequently found in the butyrate-producing *Clostridia*, especially *C. butyricum, Clostridium acetobutylicum*, and *Clostridium beijerinckii* [8,170–172].

### 3.2. Identification and assessment of VFAs-producing communities

The diversity of VFAs produced is associated with its mixed microbial community. Although metagenomic analysis has recently been used to determine microbial diversity in anaerobic processes, PCR-DGGE has advantageous because of its simplicity and high accuracy [154,173,174]. Other molecular techniques (16S rRNA-based PCR-RFLP, ribotyping, pulsed-field gel electrophoresis) have been tried for isolation of VFA-producing microorganisms [175–177]. More recently, quantitative PCR applications have also been successfully performed on specific microorganisms that produce VFA [178–180]. Since VFAs production requires a multi-stage and long-term incubation, quantitative PCR may be considered as a more appropriate method to determine which type of microorganisms is dominant at certain incubation times in AD, as well as complex communities.

### 3.3. Factors and inhibitors affecting VFAs-producing communities

Comprehending the factors and inhibitors that affect VFAs production would facilitate designing better strategies for balancing the system. Hence, more VFAs would be generated [181]. Various authors have studied the importance of physicochemical parameters such as pH, temperature, OLR, and substrate, which act as factors and inhibitors during acidogenesis in the AD process [155,182,183].

#### 3.3.1. Substrate

One of the aspects that influence the VFAs production is the substrate composition. It has been found that the carbohydrate-rich materials improve protein conversion rate and enhance the VFAs yield [184,185]. Proteins are degraded faster than lipids throughout the hydrolytic-acidogenic step [186]. It is worth noting that lipids are challenging for microorganisms to employ in fermentative situations since reductive byproducts produced from lipid fermentation would agitate the redox instability of microorganism metabolism [181]. Yin et al. [187] studied VFAs concentration generated from glucose, peptone, and lipid. The VFAs yield for glucose, peptone, and glycerol were 38.2, 32.1, and 31.1 gCOD/L, respectively. This study demonstrated that co-digestion had a synergistic impact among microorganisms. Consequently, it could ameliorate the acidogenesis process. Lignocellulosic materials prolong the hydrolysis stage by robust digestible lignin structures. Due to these recalcitrant features, the microorganism could not degrade the basic substrate (cellulose) [155,188,189]. Hence, it would lead to decreasing VFAs production rate. Several substrates contain some compounds that inhibit acidogenesis. For example, the presence of D-limonene in citrus waste has a negative impact on the AD process [190].

#### 3.3.2. pH

The biodiversity and microbial attribution in the system are affected by the profound impact of the pH. The alkaline or acidic pH conditions diminish the microbial population [191]. Due to its regulating out home for anaerobic fermentation, pH has been investigated broadly. JIang et al. [192] analyzed different pH conditions on VFAs production. They observed that pH ranging from 6.0 to 7.0 induced hydrolysis up to 20% and increased the soluble chemical oxygen demand [sCOD). These parameters doubled the VFAs production in the bioreactor. Zhang et al. [193] confirmed the pH near the neutral heads to further VFAs yield. They achieved a VFAs yield of 0.27 g VFAs/g TS compared to 0.15 g VFAs/g TS in the control group with uncontrolled pH. On the other hand, some studies demonstrated that the alkaline range could considerably increase VFAs production from sewage sludge (194]. It is recognized that alkaline pH promotes the organic material solubility and enhances their bioavailability for acidogenic microorganisms in the process [195]. In another study, the pH 4.0 and pH 12.0 inhibited the VFAs’ production [196], which can be assigned to the point that the majority of acidogenic microorganisms cannot endure actual acutely acidic (pH 3) or alkaline (pH 12) conditions [197,198].

#### 3.3.3. Temperature

Temperature plays a vital role in acidogenic fermentation owing to its straight contention in microbial growth and metabolism. Altering working temperature can change the composition of microorganisms in the microbial consortium included in acidogenesis. He et al. [199] discovered the inhibitory effect of temperature when it changed from mesophilic (35°C) to thermophilic range (55°C), led to a drop in VFAs production from 17 to 11 g/L. Similarly, later studies supported this theory as they observed a remarkable advance in acidogenesis in AD. Subsequently, increasing the temperature leads to an improvement in the degradation of organic matters [200].

#### 3.3.4. Salts and heavy metals

Salts could change the microbial community and VFAs yield in the process. He et al. [201] investigated the effect of four different concentrations on the acidogenesis stage. They reported an inhibition at NaCl concentration of 10 g/L. Likewise, Kim et al. [202] observed that a high concentration of NaCl would suppress acidogenic fermentation. Heavy metals inhibit VFAs production as they are toxic for VFAs-producing microorganisms. Yu and Fang [203] studied the toxic concentration range of Cd. They showed that the dosage over 20 mg/L inhibits acidogenesis. However, Cd dosage at 5 mg/L increased the VFAs production by 100%. All mentioned factors have a practical impact when they are in the optimum range; however, they can act as an inhibitory factor in extreme ranges and disrupt the microbial community that has a role in acidogenesis. Eventually, it leads to system failure in VFAs production.

## 4. Anaerobic digestion for hydrogen production

The demand for clean energy sources has increased in the recent past. Biohydrogen is considered a promising carbon-free fuel with many socio-economic benefits that can be a suitable source of clean energy [24,204]. Fascinatingly, hydrogen is a valuable raw material for the synthesis of chemicals (e.g. ammonia, ethanol, and aldehydes), fossil fuels, and edible oils hydrogenation [205,206]. In recent years, the AD process is underexploited to determine the appropriate configuration for hydrogen production. At the present time, 99% of hydrogen is produced from natural gas, heavy oils, coal, and electrolysis that do not help to solve environmental problems. Hence, hydrogen production via biological processes can be an environmentally friendly alternative to routine methods. Already, AD of affordable renewable feedstock and various organic wastes is the best biological way for hydrogen production because, unlike light fermentation (by phototrophic purple non-sulfur bacteria) and photosynthesis (by cyanobacteria), biohydrogen production via AD does not require expensive and complicated bioreactors and can produce hydrogen continuously from renewable sources [6,41,207–209].

**Figure 4.**
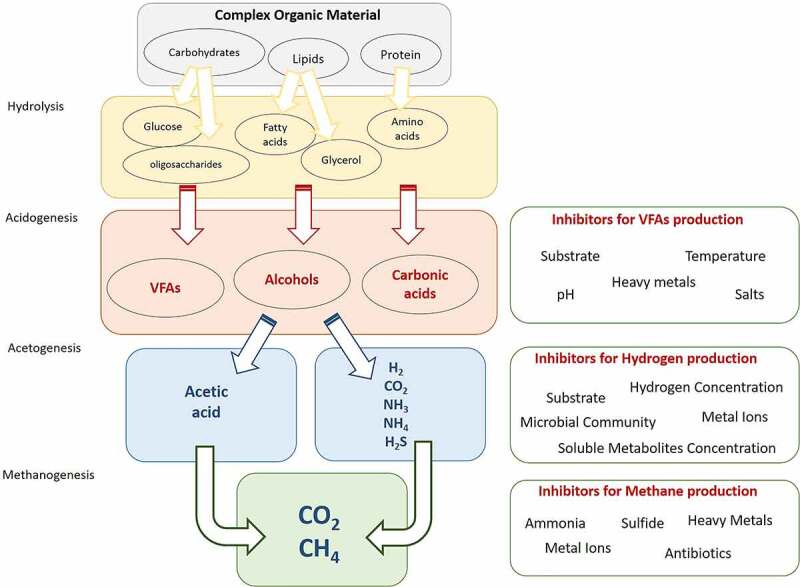
Hydrogen formation during AD. Red crosses show blocked pathways that lead to more hydrogen production. Inhibitors of each stage were shown in the right boxes.

Despite available information about the AD process, hydrogen production through the AD process is an emergent technology that still requires more attention and research to realize the economic conditions, sustainability of the process, optimal conditions for improving yield in macro-scale production, and other aspects. Figure 4 summarizes the hydrogen production pathway during AD. In addition to routine AD, two-step anaerobic digestion (TSAD) can be used for biohydrogen production. This approach consists of two different bioreactors that separate hydrogen-producing microorganisms (HPMs) and hydrogen-consuming microorganisms (HCMs), mostly *Clostridia* and hydrogenotrophic methanogens, from each other. This kind of processing facilitates bioreactor operation, and more energy yield than a one-step AD can be achieved. Because in one-step AD, only one-third of the energy content can be captured in the hydrogen, while in the TSAD process, remaining organic materials and by-products can be more converted to methane, hydrogen, or other products [210,211].

### 4.1. Microbiology of biohydrogen production

During conventional AD of organic materials, hydrogen is produced as an essential intermediate that can be assimilated via methanogenesis to produce methane. The microbial production of hydrogen is a vital response to the cellular necessity to discharge extra electrons from the biological system [212]. HPMs are responsible for biohydrogen production via DF. The theoretical yield of hydrogen strongly depends on microbial communities and their growth conditions. During DF, the enzyme hydrogenase and the protein ferredoxin (Fd) play an essential role in the production of gaseous hydrogen. The hydrogenases are usually located at accessible positions from outside by electron shuttles in the periplasm of HPMs. The Fd is an iron-sulfur protein that acts as an electron carrier at low redox potential. Inclusively, microorganisms through hydrogen production maintain the redox potential balanced. If the hydrogen production is prohibited, more reduced compounds will be formed [209].

In general, favorable conditions for hydrogen production occur when HCMs are inhibited, and HPMs get the dominant communities. HPMs have a broader pH range and can grow more rapidly than HCMs. Besides, HPMs are more resistant to harsh conditions in comparison to methanogens. Among HPMs, the best hydrogen producers belong to the anaerobic genus *Clostridium* with the capability of utilizing numerous organic substrates. However, they can exhibit various metabolic configurations based on the conditions, used substrates, and whole process design. For instance, *C. acetobutylicum* is able to shift its metabolism from hydrogen production to solventogenic metabolism under low pH, high concentrations of carbohydrates, or low growth rate of the cells [213]. *Clostridia* can be grouped into two types of proteolytic and saccharolytic based on the substrate type. However, some *Clostridia* are neither proteolytic nor saccharolytic, and some of them are both proteolytic and saccharolytic. Approximately, 70–80% of the mixed HPMs in a bioreactor belong to the genus *Clostridium*. The gram-positive, spore-forming and strictly anaerobic members of this genus can break down glucose into pyruvate and produce NADH (Nicotinamide adenine dinucleotide hydrogen) via DF. Under low partial pressure of hydrogen, the NADH molecule can be oxidized by hydrogenases that results in additional hydrogen production [214]. Produced pyruvate is further broken down to acetyl-CoA and CO_2_. Then, acetyl-CoA is transformed to acetyl phosphate as ATP and acetate excreted. During oxidation of pyruvate into acetyl-CoA by pyruvate ferredoxin oxidoreductase (PFOR), the protein Fd is reduced and then oxidized by the hydrogenase to produce gaseous hydrogen. Moreover, the NADH is oxidized which produces hydrogen under low partial pressures of hydrogen. The order of highest yields of hydrogen from VFAs is acetate>butyrate>propionate>alcohols>lactate [6,209,215,216]. In addition to *Clostridium*, the facultative anaerobic bacteria such as the genera *Enterobacter, Alcaligenes, Escherichia*, and *Citrobacter* from the phylum *Proteobacteria*, some species of *Bacillus*, some cyanobacteria (*Synechocystis*), and algal strains from the genus *Chlamydomonas* are considered as HPMs [217–219]. These microorganisms are less sensitive than *Clostridia* to oxygen; however, in the presence of oxygen, their substrate (formate) for hydrogen production degrades and, no hydrogen can be produced. The key enzyme in this kind of HPMs is pyruvate formate lyase (PFL) that reversibly converts pyruvate into formate and acetyl-CoA. Produced formate is further metabolized to hydrogen and CO_2_ [6,220,221].

The DF process can be carried out under mesophilic and thermophilic conditions. Microbial communities will vary based on the used temperature. Mesophilic DF has a high capacity for hydrogen production, but due to the production of other reduced products, hydrogen yield is low. In this process, initial inoculum originates from soils, compost, or wastewater sludge with undefined microbial communities that mainly contain facultative and obligatory anaerobic HPMs. However, undesired microorganisms such as methanogens and propionic acid bacteria (PAB) may be present in the inoculum. Hence, it is mandatory to perform a pretreatment to minimize HCMs [8]. Thermophilic DF can be performed by a broad range of thermophiles such as thermophilic species of *Clostridium* (e.g. *Clostridium thermocellum*) and *Caldicellulosiruptor* [222,223]. This process has some advantages, such as the lower risk of contamination, a wide range of utilizable substrates, and less formation of various by-products in comparison to mesophilic DF. Moreover, the thermophilic hydrogen producers are usually isolated from hot springs and hydrothermal vents with a glycoside hydrolytic activity (via extracellular hydrolases or attached cellulosome) that makes them enable to break down lignocellulosic materials [8,205].

Based on the main goal in the AD process, the operation of the digesters may vary. In the old-fashioned AD, the final goal is methane production; hence, it is desirable to utilize hydrogen for methane generation. In general, hydrogen produced in the digesters is directly assimilated by a wide variety of microorganisms included hydrogenotrophic methanogens, homoacetogens, SRBs, autotrophic denitrifiers, or iron reducers. Therefore, it is compulsory to repress the growth of HCMs. Many researchers reported methods employed to sewage sludge to dominate hydrogen-producing microbial communities [6,209]. The heat shock (80–100°C for 20–60 min) and freeze/thawing (−20/25°C cycle for 6 h) are the most routine ways for the selection of resistant HPMs due to their ability for spore formation. The pH (lower than 6.3 or higher than 7.8), radiation, electric current, aerobic stresses, kinetic selection, or use of chemicals such as acetylene, chloroform, and 2-bromoethanesulfonate (BES) can be applied to inhibit HCMs and increase the HPMs population [224,225]. However, chemicals are not added continuously because of the probable resistance of HCMs to them that result in a higher dose of inhibitors. By employing the higher dose of chemicals, HPMs may be affected that does not make it a sustainable approach for long-term usage. For kinetic selection, Yang and Shen [226] used anaerobic mixed cultures that were enriched in a chemostat for 1 month which the HRT was hold at 12 h (a short HRT); therefore, HCMs such as methanogens were washed out due to their slower growth rates in comparison to HPMs. In fact, specific growth rate (μ) is approximately 4–5 times greater for HPMs [225].

In the anaerobic digesters, some other communities rather than HPMs may benefit the hydrogen production process. The microorganisms that regulate the oxygen content and those that regulate the medium pH, such as lactic acid bacteria (LAB), are vital for hydrogen production. However, LAB may be a double-edged sword that compete with HPMs for pyruvate and inhibit them by lowering pH or producing bacteriocins. Some other bacteria can metabolize VFAs in order to prevent their accumulation in the system and provide a buffering condition [41]. Moreover, *Bacillus, Paenibacillus, Prevotella*, or *Klebsiella* can produce exopolysaccharides and form granules to increase the resistance of microbial biomass to toxic substances and prevent biomass losing [227].

### 4.2. Identification and assessment of hydrogen-producing communities

Among various molecular techniques, PCR-DGGE easily reveals and visualizes mixed microbial culture communities to analyze biohydrogen producers, so it is the most commonly applied method [228,229] (Table 5). The first step in identifying microorganisms is the isolation of genomic DNA followed by identification with standard 16s rRNA primers. Also, hydrogenase genes or their transcripts can be screened for identifying a hydrogen producer microorganism [230–232].

**Table 5.** Molecular-based methods for identification of hydrogen producers

Molecular Method	Primers	Microorganisms	References
PCR-DGGE	16S rDNA	*Thermoanaerobacterium thermosaccharolyticum*	[294]
	Universal Primer	*Clostridium* sp. (possibly *Clostridium pasteurianum*) *Klebsiella oxytoca* *Streptococcus* sp.	[295]
	16S rRNA	*Clostridium diolis* *C. butyricum* *Aneurinibacillus aneurinilyticus* *Bacillus* sp. Swine manure bacterium	[296]
	V3–16S rDNA region with *puf*M gene fragments	*Rhodopseudomonas palustris*	[297]
	16S rDNA	*C. butyricum* *C. pasteurianum* *C. tyrobutyricum* *Klebsiella pneumoniae* *Streptococcus* sp. *Pseudomonas* sp. *Bifidobacterium* *Dialister*	[298]
	Specific PCR primer set (Chis150f–ClostIr), rRNA, Clusters I and II *Clostridia*	*C. pasteurianum* *C. butyricum* *C. tyrobutyricum* *K. pneumoniae* *Dialister* *Bifidobacterium* sp.	[232]
	16S rRNA, forward primer C356F with reverse primer 517 R	*Citrobacter freundii* *Clostridium perfringens* *Lachnospiraceae* *Enterobacter cloacae*	[299]
	Primer pair EUB968F, UNIV1392R, 16S rRNA	*Clostridium stercorarium* *C. pasteurianum* *Thermoanaerobacterium saccharolyticum*	[300]
	16S rDNA	*Majority: Clostridia and Bacilli* *Desulfotomaculum putei* *Clostridium difficile* *Clostridium polysaccharolyticum* *Bacillus cereus* *Bacillus subtilis* *Bacillus licheniformis* *Dialister* sp. *Minority: Bacteroidia, Flavobacteria and Aquificae* *Prevotella buccae* *Bactericides acidifaciens* *Prevotella stercorea* *Tamlana sediment* *Sulfurihydrogenibium kristjanssonii*	[231]
	Universal Primer	*Enterobacter ludwigii* *Shigella sonnei* *Bacillus amyloliquefaciens* *Bacillus atrophaeus* *B. licheniformis* *B. subtilis* *Staphylococcus warneri* *A. faecalis* *C. freundii*	[301]
16S rDNA-based T-RFLP	PCR primer 27 F-FAM with 1492 R	*Clostridium paraputrificum* *C. butyricum* *C. acetobutylicum* *C. beijerinckii*	[302]
FISH	Oligonucleotide probes	*Clostridium*	[295]
	Oligonucleotide probes labeled with Cy3	*Clostridium* genus	[298]
Real-time PCR	16S rRNA gene	*Clostridium* spp. *Klebsiella* spp. *Streptococcus* spp. *Pseudomonas* spp. *Bifidobacterium* spp.	[230]
RT-qPCR	Hydrogenase genes of hydrogen-producing	*C. pasteurianum* *C. butyricum*	
	Hydrogenase mRNA-targeted	*Clostridium saccharobutylicum* *C. pasteurianum*	[233]

The qPCR method combines qualitative and quantitative analysis and is also a faster method that can be used for both mRNA and DNA targeting analyses. However, this method has been too limited in the determination of microorganisms that can produce hydrogen [230–235]. Similarly, other molecular techniques, such as T-RFLP and RISA, have been rarely used for identifying microorganisms (Table 5). FISH is a method generally used to determine its presence in a sample using 16S rRNA probes specific to a particular bacterial strain, species or taxon.

Recently, NGS has started a great era in microbial ecology and genomic screening studies. NGS can easily identify microbial communities of environmental samples (such as seawater, soil, wastewater, etc.) in a short time at low cost. Similarly, researches on the identification of hydrogen-producing microorganisms by the NGS method have been applied [236,237].

### 4.3. Factors and inhibitors affecting hydrogen-producing communities

Various factors that may inhibit biohydrogen production could be extensively categorized as preprocess and in-process inhibitors. Preprocess inhibitors already exist before AD. This category roofs the microflora or substrates properties. Provided, in-process inhibitors such as metal ions, hydrogen pressure, and soluble metabolites are formed over the path of the AD process. Many factors may affect HPMs, especially *Clostridia* that are very sensitive to environmental conditions. These factors include temperature, operating pH, redox potential, HRT, OLR, substrate, nutrients, hydrogen partial pressure, reactor configuration, initial inoculum, pretreatment, and mixing [6,209,238]. One of the most important factors that lead to the limitation of hydrogen production is hydrogen partial pressure. Hydrogen should be effectively removed from the system; otherwise, it accumulates up to 12–70 times in the medium and causes the ratio of NADH/NAD+ to increase that shift the cellular metabolism toward other end products [8].

#### 4.3.1. Microbial community structure

The hydrogenotrophic methanogens, homoacetogens, and SRB consume hydrogen as substrate and increase different end products such as sulfide, which inhabit the hydrogen production [239,240]. Hydrogenotrophic methanogens utilize H_2_ as the electron donor for reducing CO_2_ to produce methane, resulting in a diminished hydrogen yield [241,242]. Homoacetogens can grow autotrophically, heterotrophically, or mixotrophically on various substances to produce acetate [239,243]. Autotrophic homoacetogens support the generation of acetate by reducing CO_2_ using H_2_ as the electron donor, therefore decrease the hydrogen concentration in the process [244]. SRB use a diversity of substrates as electron donors such as H_2_ to reduce sulfate to sulfide and decrease the hydrogen yield. Furthermore, these bacteria release sulfide which has been reported to have toxic impacts on the performances and growth rates of HPMs [245].

#### 4.3.2. Substrate

It is evident from the experimental studies that overall hydrogen production could be influenced by substrate concentration [organic load) and substrate composition. Mohan et al. [246] found an improvement in hydrogen production with increasing organic load from 4.8 to 32.0 kg COD/m^3^. Afterward, it revealed a subtractive trend from 36.0 kg COD/m^3^. Although carbohydrate-rich wastes have the potential to be utilized for hydrogen production, they can produce more acid during digestion and reduce the pH range thereby preventing optimal hydrogen production (247].

One of the most effective treatments for organic waste is co-digestion that forms different organic substrates as a homogenous mixture for anaerobic digestion. Due to the synergy between substances that compensate for the nutrient deficiency in the process, providing better pH conditions and a more balanced C/N ratio, co-digestion can improve the efficiency of hydrogen production [248,249]. Nevertheless, co-digestion requires a delicate equilibrium among organic substances, since low volatile solid or high biodegradability of substrates would lead to VFAs accumulation in-process and cause inhibition in biohydrogen production [250].

#### 4.3.3. Metal Ions

Metal ions have a vital role in the AD process. They facilitate microbial metabolism, cell growth, enzyme activation, and biohydrogen production [251,252]. Metal ions could be categorized into light metal ions such as Mg^2+^, Na^+^, and Ca^2+^ and heavy metal ions such as Fe^2+^ and Ni^2+^ [116]. Nevertheless, biohydrogen production might be suppressed by high concentrations of metal ions [253]. Bao et al. [254] unveiled that the addition of 20.0 mg/L Mg^2+^ would inhibit biohydrogen production from starch. This inhibition in hydc rogen production was also confirmed by Li and Fang [253], who stated that 1600 mg/L of nickel was toxic for bioactivity of HPMs and decreased the rate of hydrogen production .

#### 4.3.4. Hydrogen concentration

The reduction of protons to hydrogen at a high concentration of H_2_ in the liquid phase is thermodynamically inappropriate, leading to oxidation of H_2_ to proton and inhibition of hydrogen production [255]. Dong et al. [256] revealed that the partial pressure of H_2_ could suppress the conversion of long-chain fatty acids into acetate and H_2_. Besides, Van Niel [257] reported that high H_2_ partial pressure causes a metabolic shift to promote acetate, ethanol, acetone, and butanol generation at the expense of H_2_.

#### 4.3.5. Soluble metabolites concentration

Soluble metabolites are also produced during AD. These metabolites include organic acids such as acetic acid, propionic acid, butyric acid, formic acid, and lactic acid, or solvents such as ethanol, acetone, or butanol [258]. The pathway is commonly categorized as acidogenesis (for organic acids’ generation) and solventogenesis (for solvents’ formation) [259]. These metabolites negatively affect hydrogen production by increasing the ionic strength and diminishing the pH of anaerobic digestion, which leads to cell death of HPMs and decreases hydrogen production [260,261]. The inhibitory threshold concentration of each factor is varied between studies, which could be assigned to the various sources and concentrations of inoculum and substrates and diverse process conditions like temperature and pH.

## 5. Anaerobic digestion for other purposes

Along with the main products of the AD process, some other by-products may produce that depends on how organic matters are broken down and which kind of microorganisms are fermenting. For example, polyhydroxyalkanoates, nisin, amino acids, solvents, phenolics, flavonoids, di/tricarboxylic acids, lactic acid, carotenoids, terpenes, or furans may form during the processing [6,41,151]. Another significant by-product that is formed during the AD of wastes is stabilized sludge (digestate) which is a suitable fertilizer for various lands such as public parks or agricultural lands. This fertilizer is more appropriate than undigested ones such as manure due to its decreased organic matters, odor, and pathogens. Moreover, the nutrient bioavailability is increased in the digested sludge [4,13,16,19,262]. Digestate is recently subjected to various trace elements recovery such as phosphorus due to its higher content in the digestate [263,264]. Moreover, digestate could be a suitable and affordable nutrient source for microalgae cultivation [265]. In addition to by-products, the configuration of the AD can be used for various purposes such as metal removal and recovery, bioremediation of recalcitrant and toxic compounds, and nitrate removal via nitrification [27]. The AD process can be employed to mitigate heavy metals pollution. In a recent study, Shi et al. [266] showed a mixed microbial consortium in an anoxic membrane batch biofilm reactor could perform methane-dependent selenate reduction with high rates. The mixed consortium was composed of methanogens and bacteria, and the reduction efficiency decreased meaningfully in the absence of each group.

### 5.1. Microbial fuel cells with anaerobic digestion

Nowadays, MFCs with the AD process are promising devices in which the biochemical energy of organic matter converts into electricity. MFCs have been increasingly applied for industrial wastewater treatment and renewable energy generation. In the MFCs, anaerobic exoelectrogenic bacteria oxidize the substrates in the anodic chamber under anoxic conditions, and the released electrons are transferred to the cathode to generate electrical energy [33,267,268]. The MFCs can work with mixed and pure cultures. Mixed cultures show excessive resistance to the interfering processes and have higher rates for substrates intake. This natural community can be obtained from activated sludge, lakes, or sediments in which various bacterial strains such as *Shewanella, Geobacter, Aeromonas, Bacteroides, Clostridium, Desulfuromonas, Alcaligenes faecalis, Enterococcus faecium*, and *Pseudomonas aeruginosa* are found. Despite many researches, the application of MFCs using AD encounters some struggles (e.g. high costs of equipment) that limit scaling up for the industry [27,41,269].

### 5.2. Rumen: a unique environment for anaerobic digestion

One of the natural ecosystems in which AD occurs is the rumen of ruminant animals such as cows, deer, and sheep. The rumen is a complicated environment with diverse microbial populations that effectively digest various compounds through intensive and coordinated activities of hydrolytic enzymes such as cellulases, esterases, or amylases and produce VFAs and biogas. Hence, rumen can be used as a model for industrial AD systems such as batch reactor (BR), plug-flow reactor (PFR), anaerobic sequencing batch reactor (ASBR), and continuous flow stirred-tank reactor (CSTR) to improve production rate, operation, and system control [270]. In addition, using rumen microbial communities in AD reactors had an increasing trend in recent years. These artificial rumens or rumen derived anaerobic digestion (RUDAD) can simulate biological processes and microbial relationships that lead to considerable development and insights for AD in the so-called rumen simulating technique (RUSITEC) [270,271]. However, there are notable differences between rumen and anaerobic digesters. For instance, in the rumen, absorption reactions take place along with hydrolysis that results in the removal of hydrolysis and fermentation products such as VFAs and affects cellulolytic activities of rumen microorganisms. Another difference is related to a process known as rumination. During rumination, semi-digested materials are vomited and chewed again for a long time. This process increases the digestible surfaces of materials to facilitate microorganism’s access and their enzymes attacks. The rumination can be compared with upstream processing carried out in the AD bioreactors [270,272]. In addition to modeling, seeding of conventional anaerobic digesters with rumen introduces microorganisms with a higher hydrolytic activity that can facilitate degradation of lignocellulosic materials and decreases pretreatment costs [273–276]. In a study, HU and Yu [277] showed cattail was effectively converted into VFAs, particularly acetate and propionate by rumen microorganisms during 125 h, as the total VFAs production was 371.9 mg/g volatile solids.

Although most microbial communities of the rumen are not cultured, omics approaches and functional analysis showed the main microbial structures of the rumen. The most important bacteria isolated from rumen belong to the genera *Fibrobacter, Ruminobacter, Bacillus, Lactobacillus, Clostridium*, and *Bifidobacterium*. In addition to bacteria, ciliate, protozoa, and anaerobic fungal genera such as *Neocallimas, Piromonas*, and *Sphaeromonas* are reported in the rumen to exhibit lignocellulolytic activity. Moreover, *Methanobacterium, Methanobrevibacter*, and *Methanomicrobium* are identified in the rumen that are responsible for methanogenesis [270,278–280]. Many factors such as sampling time, feeding time, feed composition, etc., may affect microbial communities of the rumen fluid that is used as inoculum for the anaerobic digesters. Consequently, inoculum composition can influence the final products of the anaerobic digesters [281,282]. For example, fresh rumen fluid (obtained at 3 h after feeding) had the highest microbial numbers, activity, and fermentation rate [281].

## 6. Conclusion and future perspectives

Regarding worldwide concerns about climate change, widespread pollution, and scientists’ efforts for the reduction of carbon footprint, biomass conversion technologies, especially AD are promising platforms. A brilliant prospect for the AD process is conceivable as a sustainable technology, in which the biochemical energy of organic matters can be efficiently extracted. The AD process is a low-priced and multi-purpose technology, which can use various feedstocks such as food wastes, various wastewaters, agricultural residues, sludge, manure, etc. AD is a very complex biological process, and intensive efforts are still required to determine effective factors and optimum conditions for stabilization, higher yield, and productivity of new high-value products such as hydrogen and VFAs. Moreover, some impediments influence the AD process that should be overcome to achieve the great potential of this process for bioenergy and other products production. In this way, both traditional and cutting-edge methods are necessary to attain these goals. Precise prediction, process monitoring, real-time controlling, and modeling of microbial communities’ performance in the AD system would be promising and informative approaches for improved AD operation. Additionally, to investigate microbial communities’ complexity, their structure, function, activities, and interactions during AD, omics approaches have had successful impacts in recent years. These approaches may reveal an accurate vision of microbial and viral biodiversity and the spatial organization of microbial communities in different anaerobic digesters. However, all involved species in the AD process are not known and identified completely. Providing an entire dataset of all these species, their function during four stages of AD, and their metabolic capacity may guide researchers to operate anaerobic digesters under controlled situations. Comprehensively, these findings allow us to conduct the process better and shift the various phases based on the final products desired. Furthermore, the findings can bridge among microbiologists, bioinformatics scientists, and chemical engineers to discover novel microbial communities, metabolic pathways, or products of the AD process.

## References

[1] Chew KR, Leong HY, Khoo KS, et al. Effects of anaerobic digestion of food waste on biogas production and environmental impacts: a review. Environ Chem Lett. 2021;19:2921–2939.

[2] Sekoai PT, Ghimire A, Ezeokoli OT, et al. Valorization of volatile fatty acids from the dark fermentation waste streams-A promising pathway for a biorefinery concept. Renew Sust Energ Rev. 2021;143:110971.

[3] Khanal SK. Microbiology and Biochemistry of Anaerobic Biotechnology. In: Khanal SK, editors. Anaerobic Biotechnology for Bioenergy Production: Principles and Applications. John Wiley & Sons, Inc; 2008. DOI:10.1002/9780813804545.ch2

[4] Insam H, Franke-whittle I, Goberna M. Microbes at work: from wastes to resources. Berlin Heidelberg: Springer; 2009.

[5] Kalamdhad A. Integrated approaches towards solid waste management. New Delhi, India: Springer; 2021.

[6] Khanal SK. Anaerobic biotechnology for bioenergy production: principles and applications. Iowa, USA: Wiley-blackwell; 2011.

[7] Ostrem K, Themelis NJ. Greening Waste: Anaerobic Digestion for Treating the Organic Fraction of Municipal Solid Wastes. Earth Engineering Center Columbia University; 2004.

[8] Hatti-kaul R, Mamo G, Mattiasson B. Anaerobes in biotechnology. Switzerland: Springer International Publishing; 2016.

[9] Singh L, Yousuf A, Mahapatra DM. Bioreactors: sustainable design and industrial applications in mitigation of Ghg emissions. Amsterdam, Netherlands: Elsevier Science & Technology; 2020.

[10] Weiland P. Biogas production: current state and perspectives. Appl Microbiol Biotechnol. 2010;85(4):849–860. 19777226 10.1007/s00253-009-2246-7

[11] Czatzkowska M, Harnisz M, Korzeniewska E, et al. Inhibitors of the methane fermentation process with particular emphasis on the microbiological aspect: a review. Energy Sci Eng. 2020;8:1880–1897.

[12] De Lemos Chernicharo CA. Anaerobic reactors. London, UK: IWA Publishing; 2007.

[13] Korres N, O’kiely P, Benzie JAH, et al. Bioenergy production by anaerobic digestion: using agricultural biomass and organic wastes. Abingdon, UK: Taylor & Francis; 2013.

[14] Muñoz P. Assessment of batch and semi-continuous anaerobic digestion of food waste at psychrophilic range at different food waste to inoculum ratios and organic loading rates. Waste Biomass Valorization. 2019;10(8):2119–2128.

[15] Kumar Awasthi M, Ravindran B, Sarsaiya S, et al. Metagenomics for taxonomy profiling: tools and approaches. Bioengineered. 2020;11(1):356–374. 32149573 10.1080/21655979.2020.1736238 PMC7161568

[16] Srivastava M, Srivastava N, Singh R. Bioenergy research: biomass waste to energy. Singapore: Springer Singapore; 2021.

[17] Tabatabaei M, Ghanavati H. Biogas: fundamentals, process, and operation. Switzerland: Springer International Publishing; 2018.

[18] Krakat N, Schmidt S, Scherer P. Potential impact of process parameters upon the bacterial diversity in the mesophilic anaerobic digestion of beet silage. Bioresour Technol. 2011;102(10):5692–5701. 21435870 10.1016/j.biortech.2011.02.108

[19] Merlino G, Rizzi A, Villa F, et al. Shifts of microbial community structure during anaerobic digestion of agro-industrial energetic crops and food industry byproducts. J Chem Technol Biot. 2012;87(9):1302–1311.

[20] Ivarsson M, Schnürer A, Bengtson S, et al. Anaerobic fungi: a potential source of biological H2 in the oceanic crust. Front Microbiol. 2016;7. DOI:10.3389/fmicb.2016.00674 PMC4922220 27433154

[21] Kazda M, Langer S, Bengelsdorf FR. Fungi open new possibilities for anaerobic fermentation of organic residues. Energy Sustainability Soc. 2014;4(1):1–9. DOI:10.1186/2192-0567-4-6

[22] Vinzelj J, Joshi A, Insam H, et al. Employing anaerobic fungi in biogas production: challenges & opportunities. Bioresour Technol. 2020;300:122687. 31926794 10.1016/j.biortech.2019.122687

[23] Akyol Ç, Ince O, Bozan M, et al. Fungal bioaugmentation of anaerobic digesters fed with lignocellulosic biomass: what to expect from anaerobic fungus Orpinomyces sp. Bioresour Technol. 2019;277:1–10. 30654102 10.1016/j.biortech.2019.01.024

[24] Hatti-kaul R, Mattiasson B. Anaerobes in Industrial- and Environmental Biotechnology. In: Hatti-Kaul R, Mamo G, Mattiasson B, editors. Anaerobes in Biotechnology. Switzerland: Springer International Publishing; 2016. 10.1007/10_2016_10 27277393

[25] Amin FR, Khalid H, El-mashad HM, et al. Functions of bacteria and archaea participating in the bioconversion of organic waste for methane production. SciTotal Environ. 2021;763:143007. 10.1016/j.scitotenv.2020.143007 33229086

[26] De Vrieze J, Saunders AM, HE Y, et al. Ammonia and temperature determine potential clustering in the anaerobic digestion microbiome. Water Res. 2015;75:312–323. 25819618 10.1016/j.watres.2015.02.025

[27] Narihiro T, Sekiguchi Y. Microbial communities in anaerobic digestion processes for waste and wastewater treatment: a microbiological update. Curr Opin Biotechnol. 2007;18(3):273–278. 17462878 10.1016/j.copbio.2007.04.003

[28] Biberacher M, Tum M, Günther KP, et al. Availability assessment of bioenergy and power plant location optimization: a case study for Pakistan. Renew Sust Energ Rev. 2015;42:700–711.

[29] Kainthola J, Kalamdhad AS, Goud VV. A review on enhanced biogas production from anaerobic digestion of lignocellulosic biomass by different enhancement techniques. Process Biochem. 2019;84:81–90.

[30] Kucharska K, Hołowacz I, Konopacka-łyskawa D, et al. Key issues in modeling and optimization of lignocellulosic biomass fermentative conversion to gaseous biofuels. Renewable Energy. 2018;129:384–408.

[31] Sahu N, Deshmukh S, Chandrashekhar B, et al. Optimization of hydrolysis conditions for minimizing ammonia accumulation in two-stage biogas production process using kitchen waste for sustainable process development. J Environ Chem Eng. 2017;5(3):2378–2387.

[32] Akunna JC. Anaerobic waste-wastewater treatment and biogas plants: a practical handbook. Florida, USA: CRC Press; 2018.

[33] Horan N, Yaser AZ, N WID. Anaerobic digestion processes: applications and effluent treatment. Singapore: Springer Singapore; 2018.

[34] Wang LK, Wang MHS, Hung YT. Integrated Natural Resources Research. Switzerland: Springer; 2021.

[35] Christy PM, Gopinath L, Divya D. A review on anaerobic decomposition and enhancement of biogas production through enzymes and microorganisms. Renew Sust Energ Rev. 2014;34:167–173.

[36] Joshi SJ, Deshmukh A, Sarma H. Biotechnology for sustainable environment. Singapore: Springer Singapore; 2021.

[37] Kondusamy D, Kalamdhad AS. Pre-treatment and anaerobic digestion of food waste for high rate methane production–A review. J Environ Chem Eng. 2014;2(3):1821–1830.

[38] Wang NX, LU XY, Tsang YF, et al. A comprehensive review of anaerobic digestion of organic solid wastes in relation to microbial community and enhancement process. J Sci Food Agric. 2019;99(2):507–516. 30144051 10.1002/jsfa.9315

[39] Madigan MT, Bender KS, Buckley DH, et al. Brock Biology of Microorganisms. London, UK: Pearson; 2018.

[40] Stronach SM, Rudd T, Lester JN. Anaerobic digestion processes In industrial wastewater treatment. Berlin Heidelberg: Springer; 2012.

[41] Ahamed MI, Prasad R. Recent advances in microbial degradation. Singapore: Springer; 2021.

[42] Morris BEL, Henneberger R, Huber H, et al. Microbial syntrophy: interaction for the common good. FEMS Microbiol Rev. 2013;37(3):384–406. 23480449 10.1111/1574-6976.12019

[43] Narrowe AB, Borton MA, Hoyt DW, et al. Uncovering the diversity and activity of methylotrophic methanogens in freshwater wetland soils. Msystems. 2019;4:e00320–19. 31796563 10.1128/mSystems.00320-19 PMC6890927

[44] Vanwonterghem I, Evans PN, Parks DH, et al. Methylotrophic methanogenesis discovered in the archaeal phylum verstraetearchaeota. Nat Microbiol. 2016;1(12):16170. 27694807 10.1038/nmicrobiol.2016.170

[45] Dong N, BU F, Zhou Q, et al. Performance and microbial community of hydrogenotrophic methanogenesis under thermophilic and extreme-thermophilic conditions. Bioresour Technol. 2018;266:454–462. 30005412 10.1016/j.biortech.2018.05.105

[46] LEE C, KIM J, Shin SG, et al. Quantitative and qualitative transitions of methanogen community structure during the batch anaerobic digestion of cheese-processing wastewater. Appl Microbiol Biotechnol. 2010;87:1963–1973. 20512323 10.1007/s00253-010-2685-1

[47] Gerardi MH. The microbiology of anaerobic digesters. New Jersey, USA: Wiley; 2003.

[48] Woese CR, Kandler O, Wheelis ML 1990. *NamesforLife Bacterial and Archaeal Nomenclature* [Online]. Available: https://www.namesforlife.com/search [Accessed].

[49] Jabłoński S, Rodowicz P, Łukaszewicz M. Methanogenic archaea database containing physiological and biochemical characteristics. Int J Syst Evol Microbiol. 2015;65(Pt_4):1360–1368. 25604335 10.1099/ijs.0.000065

[50] Alvarado A, Montañez-hernández LE, Palacio-molina SL, et al. Microbial trophic interactions and mcrA gene expression in monitoring of anaerobic digesters. Front Microbiol. 2014;5:597. 25429286 10.3389/fmicb.2014.00597 PMC4228917

[51] Campanaro S, Treu L, Kougias PG, et al. Metagenomic analysis and functional characterization of the biogas microbiome using high throughput shotgun sequencing and a novel binning strategy. Biotechnol Biofuels. 2016;9(1):1–17. 26839589 10.1186/s13068-016-0441-1 PMC4736482

[52] LIU WT, Chan OC, Fang HH. Characterization of microbial community in granular sludge treating brewery wastewater. Water Res. 2002;36(7):1767–1775. 12044076 10.1016/s0043-1354(01)00377-3

[53] Martins G, Salvador AF, Pereira L, et al. Methane production and conductive materials: a critical review. Environ Sci Technol. 2018;52(18):10241–10253. 30118213 10.1021/acs.est.8b01913

[54] Anderson IJ, Sieprawska-lupa M, Goltsman E, et al. Complete genome sequence of Methanocorpusculum labreanum type strain Z. Stand Genomic Sci. 2009;1(2):197–203. 21304657 10.4056/sigs.35575 PMC3035222

[55] Schnürer A, Biogas production: microbiology and technology, *Anaerobes Biotechnol*. 16 (2016), DOI:10.1186/s12896-016-0248-y 27432246

[56] St-pierre B, Wright A-DG. Comparative metagenomic analysis of bacterial populations in three full-scale mesophilic anaerobic manure digesters. Appl Microbiol Biotechnol. 2014;98(6):2709–2717. 24085391 10.1007/s00253-013-5220-3

[57] Demirel B, Yenigün O. Two‐phase anaerobic digestion processes: a review. *J* Chem Technol Biotechnol Int Res *Process* Environ *Clean* Technol. 2002;77(7):743–755. DOI:10.1002/jctb.630

[58] Westerholm M, Moestedt J, Schnürer A. Biogas production through syntrophic acetate oxidation and deliberate operating strategies for improved digester performance. Appl Energy. 2016;179:124–135.

[59] Amani TA, Nosrati MN, Sreekrishnan TRSR, Anaerobic digestion from the viewpoint of microbiological, chemical, and operational aspects — a review. Environ Rev. 2010; 18:255–278. DOI:10.1139/A10-011

[60] De Bok F, Plugge C, Stams A. Interspecies electron transfer in methanogenic propionate degrading consortia. Water Res. 2004;38(6):1368–1375. 15016514 10.1016/j.watres.2003.11.028

[61] SUN Y, Zuo J, Chen L, et al. Eubacteria and Archaea community of simultaneous methanogenesis and denitrification granular sludge. J Environ Sci (China). 2008;20(5):626–631. 18575118 10.1016/s1001-0742(08)62104-x

[62] Hoffmann RA, Garcia ML, Veskivar M, et al. Effect of shear on performance and microbial ecology of continuously stirred anaerobic digesters treating animal manure. Biotechnol Bioeng. 2008;100(1):38–48. 18023049 10.1002/bit.21730

[63] Rodrigues BC, De Mello BS, Grangeiro LC, et al. Microbial degradation in the biogas production of value-added compounds. In: Recent advances in microbial degradation. Singapore: Springer; 2021.

[64] Zhang C, SU H, Baeyens J, et al. Reviewing the anaerobic digestion of food waste for biogas production. Renew Sust Energ Rev. 2014;38:383–392.

[65] Amani T, Nosrati M, Mousavi SM, et al. Analysis of the syntrophic anaerobic digestion of volatile fatty acids using enriched cultures in a fixed-bed reactor. Water Environ Res. 2012;84(5):460–472. 22852432 10.2175/106143012x13347678384882

[66] Grotenhuis J, Smit M, Plugge C, et al. Bacteriological composition and structure of granular sludge adapted to different substrates. Appl Environ Microbiol. 1991;57(7):1942–1949. 1892385 10.1128/aem.57.7.1942-1949.1991 PMC183503

[67] Alavi-borazjani SA, Capela I, Tarelho LAC. Over-acidification control strategies for enhanced biogas production from anaerobic digestion: a review. Biomass Bioenergy. 2020;143:105833.

[68] Franke-whittle IH, Walter A, Ebner C, et al. Investigation into the effect of high concentrations of volatile fatty acids in anaerobic digestion on methanogenic communities. Waste Manage. 2014;34(11):2080–2089. 10.1016/j.wasman.2014.07.020 PMC4227971 25164858

[69] Gahlot P, Ahmed B, Tiwari SB, et al. Conductive material engineered direct interspecies electron transfer (DIET) in anaerobic digestion: mechanism and application. Environ Technol Innovation. 2020;20:101056.

[70] Shen L, Zhao Q, WU X, et al. Interspecies electron transfer in syntrophic methanogenic consortia: from cultures to bioreactors. Renew Sust Energ Rev. 2016;54:1358–1367.

[71] Kumar V, Nabaterega R, Khoei S, et al. Insight into interactions between syntrophic bacteria and archaea in anaerobic digestion amended with conductive materials. Renew Sust Energ Rev. 2021b;144:110965.

[72] Wang Z, Wang T, SI B, et al. Accelerating anaerobic digestion for methane production: potential role of direct interspecies electron transfer. Renew Sust Energ Rev. 2021b;145:111069.

[73] Summers ZM, Fogarty HE, Leang C, et al. Direct exchange of electrons within aggregates of an evolved syntrophic coculture of anaerobic bacteria. Science. 2010;330(6009):1413–1415. 21127257 10.1126/science.1196526

[74] Angelidaki I, Batstone DJ. Anaerobic Digestion: Process. In: Solid Waste Technology and Management. West Sussex, United Kingdom: Wiley; 2011. .

[75] Renslow R, Babauta J, Kuprat A, et al. Modeling biofilms with dual extracellular electron transfer mechanisms. Phys Chem Chem Phys. 2013;15(44):19262–19283. 24113651 10.1039/c3cp53759e PMC3868370

[76] Van Steendam C, Smets I, Skerlos S, et al. Improving anaerobic digestion via direct interspecies electron transfer requires development of suitable characterization methods. Curr Opin Biotechnol. 2019;57:183–190. 31174018 10.1016/j.copbio.2019.03.018

[77] Jadhav P, Muhammad N, Bhuyar P, et al. A review on the impact of conductive nanoparticles (CNPs) in anaerobic digestion: applications and limitations. Environ Technol Innovation. 2021;23:101526.

[78] Kumar M, Dutta S, YOU S, et al. A critical review on biochar for enhancing biogas production from anaerobic digestion of food waste and sludge. J Clean Prod. 2021a;305:127143.

[79] Liang J, LUO L, LI D, et al. Promoting anaerobic co-digestion of sewage sludge and food waste with different types of conductive materials: performance, stability, and underlying mechanism. Bioresour Technol. 2021b;337:125384. 34186331 10.1016/j.biortech.2021.125384

[80] Roopnarain A, Rama H, Ndaba B, et al. Unravelling the anaerobic digestion ‘black box’: biotechnological approaches for process optimization. Renew Sust Energ Rev. 2021;152:111717.

[81] Xiao L, Lichtfouse E, Senthil Kumar P. Advantage of conductive materials on interspecies electron transfer-independent acetoclastic methanogenesis: a critical review. Fuel. 2021;305:121577.

[82] Nguyen D, Khanal SK. A little breath of fresh air into an anaerobic system: how microaeration facilitates anaerobic digestion process. Biotechnol Adv. 2018;36(7):1971–1983. 30144516 10.1016/j.biotechadv.2018.08.007

[83] Holmes DE, Shrestha PM, Walker DJF, et al. Metatranscriptomic evidence for direct interspecies electron transfer between geobacter and Methanothrix species in methanogenic rice paddy soils. Appl Environ Microbiol. 2017;83(9):e00223–17. 28258137 10.1128/AEM.00223-17 PMC5394310

[84] Park M-O, Ikenaga H, Watanabe K. Phage diversity in a methanogenic digester. Microb Ecol. 2007;53(1):98–103. 17186158 10.1007/s00248-006-9053-9

[85] Pease S. An analysis of viral Metagenomes in acetate-fed anaerobic reactors. University of Washington; 2013.

[86] Zhang J, Gao Q, Zhang Q, et al. Bacteriophage–prokaryote dynamics and interaction within anaerobic digestion processes across time and space. Microbiome. 2017;5:57. 28569210 10.1186/s40168-017-0272-8 PMC5452288

[87] Tamaki H, Zhang R, Angly FE, et al. Metagenomic analysis of DNA viruses in a wastewater treatment plant in tropical climate. Environ Microbiol. 2012;14(2):441–452. 22040222 10.1111/j.1462-2920.2011.02630.x

[88] Delforno TP, Lacerda Júnior GV, Noronha MF, et al. Microbial diversity of a full-scale UASB reactor applied to poultry slaughterhouse wastewater treatment: integration of 16S rRNA gene amplicon and shotgun metagenomic sequencing. Microbiologyopen. 2017;6(3):e00443. 10.1002/mbo3.443 PMC5458456 28229558

[89] Eiserling F, Pushkin A, Gingery M, et al. Bacteriophage-like particles associated with the gene transfer agent of methanococcus voltae PS. J Gen Virol. 1999;80(Pt 12):3305–3308. 10567664 10.1099/0022-1317-80-12-3305

[90] Chien I-C, Meschke JS, Gough HL, et al. Characterization of persistent virus-like particles in two acetate-fed methanogenic reactors. PLoS One. 2013;8(11):e81040. 24278372 10.1371/journal.pone.0081040 PMC3838374

[91] Hernández S, Vives MJ. Phages in anaerobic systems. Viruses. 2020;12(10):1091. 10.3390/v12101091 PMC7599459 32993161

[92] Molnár J, Magyar B, Schneider G, et al. Identification of a novel archaea virus, detected in hydrocarbon polluted Hungarian and Canadian samples. PLOS ONE. 2020;15(4):e0231864. 32302368 10.1371/journal.pone.0231864 PMC7164591

[93] Calusinska M, Marynowska M, Goux X, et al. Analysis of ds DNA and RNA viromes in methanogenic digesters reveals novel viral genetic diversity. Environ Microbiol. 2016;18(4):1162–1175. 26568175 10.1111/1462-2920.13127 PMC7163765

[94] Heyer R, Schallert K, Siewert C, et al. Metaproteome analysis reveals that syntrophy, competition, and phage-host interaction shape microbial communities in biogas plants. Microbiome. 2019;7(1):69. 31029164 10.1186/s40168-019-0673-y PMC6486700

[95] Runa V, Wenk J, Bengtsson S, et al. Bacteriophages in biological wastewater treatment systems: occurrence, characterization, and function. Front Microbiol. 2021;12:2708. 10.3389/fmicb.2021.730071 PMC8600467 34803947

[96] Tang X, Zhou M, FAN C, et al. Benzyl butyl phthalate activates prophage, threatening the stable operation of waste activated sludge anaerobic digestion. SciTotal Environ. 2021;768:144470. 10.1016/j.scitotenv.2020.144470 33454470

[97] Epstein SS. The phenomenon of microbial uncultivability. Curr Opin Microbiol. 2013;16(5):636–642. 24011825 10.1016/j.mib.2013.08.003

[98] Nakamura K, Tamaki H, Kang MS, et al. A six-well plate method: less laborious and effective method for cultivation of obligate anaerobic microorganisms. Microbes Environ. 2011;26(4):301–306. 21685714 10.1264/jsme2.me11120

[99] Huang L-N, Zhou H, Chen Y-Q, et al. Diversity and structure of the archaeal community in the leachate of a full-scale recirculating landfill as examined by direct 16S rRNA gene sequence retrieval. FEMS Microbiol Lett. 2002;214(2):235–240. 12351237 10.1111/j.1574-6968.2002.tb11353.x

[100] Wirth R, Kovács E, Maróti G, et al. Characterization of a biogas-producing microbial community by short-read next generation DNA sequencing. Biotechnol Biofuels. 2012;5(1):41. 22673110 10.1186/1754-6834-5-41 PMC3395570

[101] Friedrich MW. Methyl‐Coenzyme m reductase genes: unique functional markers for Methanogenic and anaerobic methane‐oxidizing archaea. In: Methods in enzymology. Vol. 397. California, USA: Academic Press; 2005. 10.1016/S0076-6879(05)97026-2 16260307

[102] Chin K-J, Lukow T, Conrad R. Effect of temperature on structure and function of the Methanogenic archaeal community in an anoxic rice field soil. Appl Environ Microbiol. 1999;65(6):2341–2349. 10347011 10.1128/aem.65.6.2341-2349.1999 PMC91346

[103] Lueders T, Friedrich M. Archaeal population dynamics during sequential reduction processes in rice field soil. Appl Environ Microbiol. 2000;66(7):2732–2742. 10877762 10.1128/aem.66.7.2732-2742.2000 PMC92067

[104] Hallam Steven J, Girguis Peter R, Preston Christina M, et al. Identification of methyl coenzyme M reductase A (mcrA) genes associated with methane-oxidizing archaea. Appl Environ Microbiol. 2003;69(9):5483–5491. 12957937 10.1128/AEM.69.9.5483-5491.2003 PMC194966

[105] Lehmacher A, Klenk H-P. Characterization and phylogeny of mcrII, a gene cluster encoding an isoenzyme of methyl coenzyme M reductase from hyperthermophilic Methanothermus fervidus. Mol Gen Genet MGG. 1994;243(2):198–206. 8177216 10.1007/BF00280317

[106] Nölling J, Elfner A, Palmer JR, et al. Phylogeny of Methanopyrus kandleri based on methyl coenzyme M reductase operons. Int J Syst Evol Microbiol. 1996;46:1170–1173. 10.1099/00207713-46-4-1170 8863453

[107] Juottonen H, Galand PE, Yrjälä K. Detection of methanogenic Archaea in peat: comparison of PCR primers targeting the mcrA gene. Res Microbiol. 2006;157(10):914–921. 17070673 10.1016/j.resmic.2006.08.006

[108] Lueders T, Chin K-J, Conrad R, et al. Molecular analyses of methyl-coenzyme M reductase α-subunit (mcrA) genes in rice field soil and enrichment cultures reveal the methanogenic phenotype of a novel archaeal lineage. Environ Microbiol. 2001;3(3):194–204. 11321536 10.1046/j.1462-2920.2001.00179.x

[109] Nunoura T, Oida H, Miyazaki J, et al. Quantification of mcrA by fluorescent PCR in methanogenic and methanotrophic microbial communities. FEMS Microbiol Ecol. 2008;64(2):240–247. 18318714 10.1111/j.1574-6941.2008.00451.x

[110] Woraruthai T, Kunno J, Pongsopon M, et al. Identification and cultivation of hydrogenotrophic methanogens from palm oil mill effluent for high methane production. Int J Energy Res. 2020;44(13):10058–10070.

[111] Pohland F, Ghosh S. Developments in anaerobic stabilization of organic wastes-the two-phase concept. Environ Lett. 1971;1(4):255–266. DOI:10.1080/00139307109434990 5141417

[112] Harirchi S, Etemadifar Z, Yazdian F, et al. Efficacy of polyextremophilic Aeribacillus pallidus on bioprocessing of beet vinasse derived from ethanol industries. Bioresour Technol. 2020;313:123662. 32563794 10.1016/j.biortech.2020.123662

[113] Hosseini Koupaie E, Dahadha S, Bazyar Lakeh AA, et al. Enzymatic pretreatment of lignocellulosic biomass for enhanced biomethane production-A review. J Environ Manage. 2019;233:774–784. 30314871 10.1016/j.jenvman.2018.09.106

[114] Mudhoo A. Biogas production: pretreatment methods in anaerobic digestion. New Jersey, USA: Wiley; 2012.

[115] Tabatabaei M, Aghbashlo M, Valijanian E, et al. A comprehensive review on recent biological innovations to improve biogas production, Part 1: upstream strategies. Renewable Energy. 2020a;146:1204–1220.

[116] Chen Y, Cheng JJ, Creamer KS. Inhibition of anaerobic digestion process: a review. Bioresour Technol. 2008;99(10):4044–4064. 17399981 10.1016/j.biortech.2007.01.057

[117] Hejnfelt A, Angelidaki I. Anaerobic digestion of slaughterhouse by-products. Biomass Bioenergy. 2009;33(8):1046–1054.

[118] Kayhanian M. Ammonia inhibition in high-solids biogasification: an overview and practical solutions. Environ Technol. 1999;20(4):355–365.

[119] Christou ML, Vasileiadis S, Kalamaras SD, et al. Ammonia-induced inhibition of manure-based continuous biomethanation process under different organic loading rates and associated microbial community dynamics. Bioresour Technol. 2021;320:124323. 33157441 10.1016/j.biortech.2020.124323

[120] Gallert C, Bauer S, Winter J. Effect of ammonia on the anaerobic degradation of protein by a mesophilic and thermophilic biowaste population. Appl Microbiol Biotechnol. 1998;50(4):495–501. 9830101 10.1007/s002530051326

[121] Jiang Y, Mcadam E, Zhang Y, et al. Ammonia inhibition and toxicity in anaerobic digestion: a critical review. Journal of Water Process Engineering. 2019;32:100899.

[122] LIU C-F, Yuan X-Z, Zeng G-M, et al. Prediction of methane yield at optimum pH for anaerobic digestion of organic fraction of municipal solid waste. Bioresour Technol. 2008;99(4):882–888. 17369040 10.1016/j.biortech.2007.01.013

[123] Molnar L, Bartha I. Factors influencing solid-state anaerobic digestion. *Biol wastes*. 1989;28(1):15–24. DOI:10.1016/0269-7483(89)90045-1

[124] Wittmann C, Zeng A-P, Deckwer W-D. Growth inhibition by ammonia and use of a pH-controlled feeding strategy for the effective cultivation of mycobacterium chlorophenolicum. Appl Microbiol Biotechnol. 1995;44(3–4):519–525. 8597555 10.1007/BF00169954

[125] Borja R, Sánchez E, Duran M. Effect of the clay mineral zeolite on ammonia inhibition of anaerobic thermophilic reactors treating cattle manure. J Environ Sci *Health Part A*. 1996a;31:479–500.

[126] Robbins J, Gerhardt S, Kappel T. Effects of total ammonia on anaerobic digestion and an example of digestor performance from cattle manure-protein mixtures. Biol *Wastes*. 1989;27(1):1–14. DOI:10.1016/0269-7483(89)90026-8

[127] Angelidaki I, Ahring B. Anaerobic thermophilic digestion of manure at different ammonia loads: effect of temperature. Water Res. 1994;28(3):727–731.

[128] Kroeker E, Schulte D, Sparling A, et al. Anaerobic treatment process stability. Journal (Water Pollution Control Federation). 1979;51(4) :718–727.

[129] LIU T, Sung S. Ammonia inhibition on thermophilic aceticlastic methanogens. Water Sci Technol. 2002;45(10):113–120. 12188530

[130] Bujoczek G, Oleszkiewicz J, Sparling R, et al. High solid anaerobic digestion of chicken manure. *J* Agric Eng Res. 2000;76(1):51–60. DOI:10.1006/jaer.2000.0529

[131] Sung S, LIU T. Ammonia inhibition on thermophilic anaerobic digestion. Chemosphere. 2003;53(1):43–52. 12892665 10.1016/S0045-6535(03)00434-X

[132] Hansen KH, Angelidaki I, Ahring BK. Improving thermophilic anaerobic digestion of swine manure. Water Res. 1999;33(8):1805–1810.

[133] Borja R, Sánchez E, Weiland P. Influence of ammonia concentration on thermophilic anaerobic digestion of cattle manure in upflow anaerobic sludge blanket (UASB) reactors. Process Biochem. 1996b;31(5):477–483.

[134] Parkin G, Speece R, and Yang C, et al. Response of methane fermentation systems to industrial toxicants. Journal (Water Pollution Control Federation). 1983;55(1):44–53.

[135] Hendriksen HV, Ahring BK. Effects of ammonia on growth and morphology of thermophilic hydrogen-oxidizing methanogenic bacteria. FEMS Microbiol Lett. 1991;85(3):241–245.

[136] Peris Serrano R. Biogas process simulation using Aspen Plus. Master, Syddansk Universitet; 2010.

[137] Oleszkiewicz J, Marstaller T, Mccartney D. Effects of pH on sulfide toxicity to anaerobic processes. Environ Technol. 1989;10:815–822.

[138] Harada H, Uemura S, Momonoi K. Interaction between sulfate-reducing bacteria and methane-producing bacteria in UASB reactors fed with low strength wastes containing different levels of sulfate. Water Res. 1994;28(2):355–367.

[139] Colleran E, Pender S, Philpott U, et al. Full-scale and laboratory-scale anaerobic treatment of citric acid production wastewater. Biodegradation. 1998;9(3/4):233–245. 10022067 10.1023/a:1008389722892

[140] Grady Jr CL, Daigger GT, Love NG, et al. Biological wastewater treatment. London, UK: CRC press; 2011.

[141] Soto M, Méndez R, Lema J. Methanogenic and non-methanogenic activity tests. Theoretical basis and experimental set up. Water Res. 1993;27(8):1361–1376.

[142] Yerkes D, Boonyakitsombut S, Speece R. Antagonism of sodium toxicity by the compatible solute betaine in anaerobic methanogenic systems. Water Sci Technol. 1997;36(6–7):15–24.

[143] Vallee BL, Ulmer DD. Biochemical effects of mercury, cadmium, and lead. Annu Rev Biochem. 1972;41(1):91–128. 4570963 10.1146/annurev.bi.41.070172.000515

[144] Kohanski MA, Dwyer DJ, Hayete B, et al. A common mechanism of cellular death induced by bactericidal antibiotics. Cell. 2007;130(5):797–810. 17803904 10.1016/j.cell.2007.06.049

[145] Cetecioglu Z, Ince B, Orhon D, et al. Acute inhibitory impact of antimicrobials on acetoclastic methanogenic activity. Bioresour Technol. 2012;114:109–116. 22459958 10.1016/j.biortech.2012.03.020

[146] Chen H, Zeng X, Zhou Y, et al. Influence of roxithromycin as antibiotic residue on volatile fatty acids recovery in anaerobic fermentation of waste activated sludge. J Hazard Mater. 2020;394:122570. 32244145 10.1016/j.jhazmat.2020.122570

[147] Tian Z, Zhang Y, Yang M. Chronic impacts of oxytetracycline on mesophilic anaerobic digestion of excess sludge: inhibition of hydrolytic acidification and enrichment of antibiotic resistome. Environ Pollut. 2018;238:1017–1026. 29449116 10.1016/j.envpol.2018.02.023

[148] Bruni C, Foglia A, Eusebi AL, et al. Targeted bio-based volatile fatty acid production from waste streams through anaerobic fermentation: link between process parameters and operating scale. ACS Sustain Chem Eng. 2021;9(30):9970–9987.

[149] Wainaina S, Awasthi MK, Horváth IS, et al. Anaerobic digestion of food waste to volatile fatty acids and hydrogen at high organic loading rates in immersed membrane bioreactors. Renewable Energy. 2020;152:1140–1148.

[150] Mwene-mbeja TM, Dufour A, Lecka J, et al. Enzymatic reactions in the production of biomethane from organic waste. Enzyme Microb Technol. 2020;132:109410. 31731967 10.1016/j.enzmictec.2019.109410

[151] Hunter SM, Blanco E, Borrion A. Expanding the anaerobic digestion map: a review of intermediates in the digestion of food waste. SciTotal Environ. 2021;767:144265. 10.1016/j.scitotenv.2020.144265 33422959

[152] Kumar G, Ponnusamy VK, Bhosale RR, et al. A review on the conversion of volatile fatty acids to polyhydroxyalkanoates using dark fermentative effluents from hydrogen production. Bioresour Technol. 2019;287:121427. 31104939 10.1016/j.biortech.2019.121427

[153] LÜ F, Wang Z, Zhang H, et al. Anaerobic digestion of organic waste: recovery of value-added and inhibitory compounds from liquid fraction of digestate. Bioresour Technol. 2021;333:125196. 33901909 10.1016/j.biortech.2021.125196

[154] Qin S, Wainaina S, LIU H, et al. Microbial dynamics during anaerobic digestion of sewage sludge combined with food waste at high organic loading rates in immersed membrane bioreactors. Fuel. 2021;303:121276. 10.1016/j.biortech.2021.125296 34022478

[155] SUN J, Zhang L, LOH K-C. Review and perspectives of enhanced volatile fatty acids production from acidogenic fermentation of lignocellulosic biomass wastes. Bioresources Bioprocess. 2021;8(1):68. 10.1186/s40643-021-00420-3 PMC10992391 38650255

[156] Bhatia SK, Yang Y-H. Microbial production of volatile fatty acids: current status and future perspectives. Rev Environ Sci Bio/Technol. 2017;16(2):327–345.

[157] Feng K, LI H, Zheng C. Shifting product spectrum by pH adjustment during long-term continuous anaerobic fermentation of food waste. Bioresour Technol. 2018;270:180–188. 30218934 10.1016/j.biortech.2018.09.035

[158] Joubert W, Britz T. Characterization of aerobic, facultative anaerobic, and anaerobic bacteria in an acidogenic phase reactor and their metabolite formation. Microb Ecol. 1987;13(2):159–168. 24213213 10.1007/BF02011251

[159] Hania WB, Bouanane-darenfed A, Cayol J-L, et al. Reclassification of anaerobaculum mobile, anaerobaculum thermoterrenum, anaerobaculum hydrogeniformans as acetomicrobium mobile comb. Nov., acetomicrobium thermoterrenum comb. Nov. and acetomicrobium hydrogeniformans comb. Nov., respectively, and emendation of the genus acetomicrobium. Int J Syst Evol Microbiol. 2016;66(3):1506–1509. 26791251 10.1099/ijsem.0.000910

[160] Merli G, Becci A, Amato A, et al. Acetic acid bioproduction: the technological innovation change. SciTotal Environ. 2021;798:149292. 10.1016/j.scitotenv.2021.149292 34375263

[161] Nayak J, PAL P. Transforming waste cheese-whey into acetic acid through a continuous membrane-integrated hybrid process. Ind Eng Chem Res. 2013;52(8):2977–2984.

[162] Noman AE, Al-barha NS, Sharaf -A-AM, et al. A novel strain of acetic acid bacteria Gluconobacter oxydans FBFS97 involved in riboflavin production. Sci Rep. 2020;10(1):1–17. 32782276 10.1038/s41598-020-70404-4 PMC7419552

[163] Tang I-C, Yang S-T, Okos MR. Acetic acid production from whey lactose by the co-culture of streptococcus lactis and clostridium formicoaceticum. Appl Microbiol Biotechnol. 1988;28(2):138–143.

[164] Wang Z, YAN M, Chen X, et al. Mixed culture of Saccharomyces cerevisiae and Acetobacter pasteurianus for acetic acid production. Biochem Eng J. 2013;79:41–45.

[165] Huang YL, Mann K, Novak JM, et al. Acetic acid production from fructose by clostridium formicoaceticum immobilized in a fibrous-Bed bioreactor. Biotechnol Prog. 1998;14(5):800–806. 9758672 10.1021/bp980077f

[166] Ragsdale SW, Pierce E. Acetogenesis and the Wood–Ljungdahl pathway of CO2 fixation. Biochim Biophys Acta Proteins Proteom. 2008;1784:1873–1898. 10.1016/j.bbapap.2008.08.012 PMC2646786 18801467

[167] DU G, LIU L, Chen J. White biotechnology for organic acids. In: Pandey A, Höfer R, Taherzadeh M, et al., editors. Industrial biorefineries & white biotechnology. Amsterdam: Elsevier; 2015. p. 409–444.

[168] Wainaina S, Lukitawesa, Kumar Awasthi M, Taherzadeh MJ. Bioengineering of anaerobic digestion for volatile fatty acids, hydrogen or methane production: a critical review. Bioengineered. 2019a;10(1):437–458. 31570035 10.1080/21655979.2019.1673937 PMC6802927

[169] Yang ST, El-ensashy H, Thongchul N. Bioprocessing technologies in biorefinery for sustainable production of fuels, chemicals, and polymers. New Jersey, USA: Wiley; 2013.

[170] Atasoy M, Owusu-agyeman I, Plaza E, et al. Bio-based volatile fatty acid production and recovery from waste streams: current status and future challenges. Bioresour Technol. 2018;268:773–786. 30030049 10.1016/j.biortech.2018.07.042

[171] Duncan SH, Barcenilla A, Stewart CS, et al. Acetate utilization and butyryl coenzyme A (CoA):acetate-CoA transferase in butyrate-producing bacteria from the human large intestine. Appl Environ Microbiol. 2002;68(10):5186–5190. 12324374 10.1128/AEM.68.10.5186-5190.2002 PMC126392

[172] Jang Y-S, WOO HM, IM JA, et al. Metabolic engineering of Clostridium acetobutylicum for enhanced production of butyric acid. Appl Microbiol Biotechnol. 2013;97(21):9355–9363. 24013291 10.1007/s00253-013-5161-x

[173] Jankowska E, Duber A, Chwialkowska J, et al. Conversion of organic waste into volatile fatty acids–The influence of process operating parameters. Chem Eng J. 2018;345:395–403.

[174] Tsapekos P, Kougias P, Treu L, et al. Process performance and comparative metagenomic analysis during co-digestion of manure and lignocellulosic biomass for biogas production. Appl Energy. 2017;185:126–135.

[175] Jie W, Peng Y, REN N, et al. Volatile fatty acids (VFAs) accumulation and microbial community structure of excess sludge (ES) at different pHs. Bioresour Technol. 2014;152:124–129. 24291313 10.1016/j.biortech.2013.11.011

[176] Rossl F, Torriani S, Dellaglio F. Identification and clustering of dairy propionibacteria by RAPD‐PCR and CGE‐REA methods. J Appl Microbiol. 1998;85(6):956–964. 9871315 10.1111/j.1365-2672.1998.tb05259.x

[177] Wang X, LI X, Zhao C, et al. Correlation between composition of the bacterial community and concentration of volatile fatty acids in the rumen during the transition period and ketosis in dairy cows. Appl Environ Microbiol. 2012;78(7):2386–2392. 22267666 10.1128/AEM.07545-11 PMC3302620

[178] Atasoy M, Cetecioglu Z. Butyric acid dominant volatile fatty acids production: bio-augmentation of mixed culture fermentation by clostridium butyricum. J Environ Chem Eng. 2020;8(6):104496.

[179] LUO J, Huang W, Zhang Q, et al. Distinct effects of hypochlorite types on the reduction of antibiotic resistance genes during waste activated sludge fermentation: insights of bacterial community, cellular activity, and genetic expression. J Hazard Mater. 2021;403:124010. 33265039 10.1016/j.jhazmat.2020.124010

[180] Wang L, Zhang G, LI Y, et al. Effects of high forage/concentrate diet on volatile fatty acid production and the microorganisms involved in VFA production in cow rumen. Animals. 2020;10(2):223. 10.3390/ani10020223 PMC7070707 32019152

[181] Wainaina S, Parchami M, Mahboubi A, et al. Food waste-derived volatile fatty acids platform using an immersed membrane bioreactor. Bioresour Technol. 2019b;274:329–334. 30529480 10.1016/j.biortech.2018.11.104

[182] Chen Y, Jiang X, Xiao K, et al. Enhanced volatile fatty acids (VFAs) production in a thermophilic fermenter with stepwise pH increase–Investigation on dissolved organic matter transformation and microbial community shift. Water Res. 2017;112:261–268. 28178608 10.1016/j.watres.2017.01.067

[183] LIU H, Xiao H, YIN B, et al. Enhanced volatile fatty acid production by a modified biological pretreatment in anaerobic fermentation of waste activated sludge. Chem Eng J. 2016;284:194–201.

[184] Chen Y, LUO J, YAN Y, et al. Enhanced production of short-chain fatty acid by co-fermentation of waste activated sludge and kitchen waste under alkaline conditions and its application to microbial fuel cells. Appl Energy. 2013;102:1197–1204.

[185] Feng L, Chen Y, Zheng X. Enhancement of waste activated sludge protein conversion and volatile fatty acids accumulation during waste activated sludge anaerobic fermentation by carbohydrate substrate addition: the effect of pH. Environ Sci Technol. 2009;43(12):4373–4380. 19603649 10.1021/es8037142

[186] LEE J, Koo T, HAN G, et al. Anaerobic digestion of cattle offal: protein and lipid-rich substrate degradation and population dynamics of acidogens and methanogens. Bioprocess Biosyst Eng. 2015;38(12):2349–2360. 26376817 10.1007/s00449-015-1470-z

[187] YIN J, YU X, Wang K, et al. Acidogenic fermentation of the main substrates of food waste to produce volatile fatty acids. Int J Hydrogen Energy. 2016;41:21713–21720.

[188] Jankowska E, Chwialkowska J, Stodolny M, et al. Volatile fatty acids production during mixed culture fermentation–The impact of substrate complexity and pH. Chem Eng J. 2017;326:901–910.

[189] Tabatabaei M, Aghbashlo M, Valijanian E, et al. A comprehensive review on recent biological innovations to improve biogas production, Part 2: mainstream and downstream strategies. Renewable Energy. 2020b;146:1392–1407.

[190] Kurniawan T, Hanifah I, Wikandari R, et al. Semi-continuous reverse membrane bioreactor in two-stage anaerobic digestion of citrus waste. Materials. 2018;11(8):1341. 10.3390/ma11081341 PMC6119998 30072666

[191] LIU H, Wang J, LIU X, et al. Acidogenic fermentation of proteinaceous sewage sludge: effect of pH. Water Res. 2012;46(3):799–807. 22176743 10.1016/j.watres.2011.11.047

[192] Jiang J, Zhang Y, LI K, et al. Volatile fatty acids production from food waste: effects of pH, temperature, and organic loading rate. Bioresour Technol. 2013;143:525–530. 23831761 10.1016/j.biortech.2013.06.025

[193] Zhang B, Zhang L, Zhang S, et al. The influence of pH on hydrolysis and acidogenesis of kitchen wastes in two-phase anaerobic digestion. Environ Technol. 2005;26:329–340. 15881029 10.1080/09593332608618563

[194] Yuan H, Chen Y, Zhang H, et al. Improved bioproduction of short-chain fatty acids (SCFAs) from excess sludge under alkaline conditions. Environ Sci Technol. 2006;40:2025–2029. 16570631 10.1021/es052252b

[195] Cai M, LIU J, WEI Y. Enhanced biohydrogen production from sewage sludge with alkaline pretreatment. Environ Sci Technol. 2004;38(11):3195–3202. 15224755 10.1021/es0349204

[196] Wang K, YIN J, Shen D, et al. Anaerobic digestion of food waste for volatile fatty acids (VFAs) production with different types of inoculum: effect of pH. Bioresour Technol. 2014;161:395–401. 24727700 10.1016/j.biortech.2014.03.088

[197] Dinamarca S, Aroca G, Chamy R, et al. The influence of pH in the hydrolytic stage of anaerobic digestion of the organic fraction of urban solid waste. Water Sci Technol. 2003;48(6):249–254. 14640225

[198] Singhania RR, Patel AK, Christophe G, et al. Biological upgrading of volatile fatty acids, key intermediates for the valorization of biowaste through dark anaerobic fermentation. Bioresour Technol. 2013;145:166–174. 23339903 10.1016/j.biortech.2012.12.137

[199] HE M, SUN Y, Zou D, et al. Influence of temperature on hydrolysis acidification of food waste. Procedia Environ Sci. 2012;16:85–94.

[200] Dichtl N. Thermophilic and mesophilic (two‐stage) anaerobic digestion. Water Environ J. 1997;11(2):98–104. DOI:10.1111/j.1747-6593.1997.tb00098.x

[201] HE X, YIN J, LIU J, et al. Characteristics of acidogenic fermentation for volatile fatty acid production from food waste at high concentrations of NaCl. Bioresour Technol. 2019;271:244–250. 30273828 10.1016/j.biortech.2018.09.116

[202] KIM D-H, KIM S-H, Shin H-S. Sodium inhibition of fermentative hydrogen production. Int J Hydrogen Energy. 2009;34(8):3295–3304.

[203] YU H, Fang HH. Inhibition by chromium and cadmium of anaerobic acidogenesis. Water Sci Technol. 2001;43:267–274. 11443972

[204] Chai WS, BAO Y, Jin P, et al. A review on ammonia, ammonia-hydrogen and ammonia-methane fuels. Renew Sust Energ Rev. 2021;147:111254.

[205] Nissilä ME, LAY C-H, Puhakka JA. Dark fermentative hydrogen production from lignocellulosic hydrolyzates – a review. Biomass Bioenergy. 2014;67:145–159.

[206] Sivagurunathan P, Kumar G, Bakonyi P, et al. A critical review on issues and overcoming strategies for the enhancement of dark fermentative hydrogen production in continuous systems. Int J Hydrogen Energy. 2016;41(6):3820–3836.

[207] Hallenbeck PC, Benemann JR. Biological hydrogen production; fundamentals and limiting processes. Int J Hydrogen Energy. 2002;27(11–12):1185–1193.

[208] Hawkins AS, HAN Y, Lian H, et al. Extremely thermophilic routes to microbial electrofuels. ACS Catal. 2011;1(9):1043–1050.

[209] Ruggeri B, Tommasi T, Sanfilippo S. BioH2 & Bioch4 through anaerobic digestion: from research to full-scale applications. London, UK: Springer; 2015.

[210] De La Rubia MA, Raposo F, Rincón B, et al. Evaluation of the hydrolytic–acidogenic step of a two-stage mesophilic anaerobic digestion process of sunflower oil cake. Bioresour Technol. 2009;100(18):4133–4138. 19414248 10.1016/j.biortech.2009.04.001

[211] LIN C-Y, Chai WS, LAY C-H, et al. Optimization of hydrolysis-acidogenesis phase of swine manure for biogas production using two-stage anaerobic fermentation. Processes. 2021;9(8):1324.

[212] Moscoviz R, Toledo-alarcón J, Trably E, et al. Electro-fermentation: how to drive fermentation using electrochemical systems. Trends Biotechnol. 2016;34(11):856–865. 27178018 10.1016/j.tibtech.2016.04.009

[213] Lütke-eversloh T, Bahl H. Metabolic engineering of Clostridium acetobutylicum: recent advances to improve butanol production. Curr Opin Biotechnol. 2011;22(5):634–647. 21377350 10.1016/j.copbio.2011.01.011

[214] Ramírez-morales JE, Tapia-venegas E, Toledo-alarcón J, et al. Simultaneous production and separation of biohydrogen in mixed culture systems by continuous dark fermentation. Water Sci Technol. 2015;71(9):1271–1285. 25945842 10.2166/wst.2015.104

[215] Mandal B, Nath K, DAS D. Improvement of biohydrogen production under decreased partial pressure of H2 by Enterobacter cloacae. Biotechnol Lett. 2006;28(11):831–835. 16786249 10.1007/s10529-006-9008-8

[216] Nath K, DAS D. Improvement of fermentative hydrogen production: various approaches. Appl Microbiol Biotechnol. 2004;65(5):520–529. 15378294 10.1007/s00253-004-1644-0

[217] Goyal Y, Kumar M, Gayen K. Metabolic engineering for enhanced hydrogen production: a review. Can J Microbiol. 2013;59(2):59–78. 23461513 10.1139/cjm-2012-0494

[218] OH Y-K, Seol E-H, KIM JR, et al. Fermentative biohydrogen production by a new chemoheterotrophic bacterium citrobacter sp. Y19. Int J Hydrogen Energy. 2003;28(12):1353–1359.

[219] REN N, CAO G, Wang A, et al. Dark fermentation of xylose and glucose mix using isolated Thermoanaerobacterium thermosaccharolyticum W16. Int J Hydrogen Energy. 2008;33(21):6124–6132.

[220] Tapia-venegas E, Ramirez-morales JE, Silva-illanes F, et al. Biohydrogen production by dark fermentation: scaling-up and technologies integration for a sustainable system. Rev Environ Sci Bio/Technol. 2015;14(4):761–785.

[221] ZHU H, Béland M. Evaluation of alternative methods of preparing hydrogen producing seeds from digested wastewater sludge. Int J Hydrogen Energy. 2006;31:1980–1988.

[222] Cappelletti M, Zannoni D, Postec A, et al. Members of the order Thermotogales: from microbiology to hydrogen production. In: Zannoni D, De Philippis R. Microbial bioenergy: hydrogen production. Dordrecht, Netherlands: Springer; 2014. p. 197–224.

[223] Ivanova G, Rakhely G, Kovacs KL. Hydrogen production from biopolymers by Caldicellulosiruptor saccharolyticus and stabilization of the system by immobilization. Int J Hydrogen Energy. 2008;33(23):6953–6961.

[224] HU B, Chen S. Pretreatment of methanogenic granules for immobilized hydrogen fermentation. Int J Hydrogen Energy. 2007;32(15):3266–3273.

[225] Petre M. Environmental biotechnology: new approaches and prospective applications. London, UK: IntechOpen; 2013.

[226] Yang H, Shen J. Effect of ferrous iron concentration on anaerobic bio-hydrogen production from soluble starch. Int J Hydrogen Energy. 2006;31(15):2137–2146.

[227] Cabrol L, Marone A, Tapia-venegas E, et al. Microbial ecology of fermentative hydrogen producing bioprocesses: useful insights for driving the ecosystem function. FEMS Microbiol Rev. 2017;41(2):158–181. 28364728 10.1093/femsre/fuw043

[228] Kumar G, Mathimani T, Sivaramakrishnan R, et al. Application of molecular techniques in biohydrogen production as a clean fuel. SciTotal Environ. 2020;722:137795. 10.1016/j.scitotenv.2020.137795 32208247

[229] Tolvanen KE, Karp MT. Molecular methods for characterizing mixed microbial communities in hydrogen-fermenting systems. Int J Hydrogen Energy. 2011;36(9):5280–5288.

[230] Cheng C-H, Hsu S-C, WU C-H, et al. Quantitative analysis of microorganism composition in a pilot-scale fermentative biohydrogen production system. Int J Hydrogen Energy. 2011;36(21):14153–14161.

[231] Goud RK, Raghavulu SV, Mohanakrishna G, et al. Predominance of bacilli and clostridia in microbial community of biohydrogen producing biofilm sustained under diverse acidogenic operating conditions. Int J Hydrogen Energy. 2012;37(5):4068–4076.

[232] Hung C-H, Cheng C-H, Cheng L-H, et al. Application of Clostridium-specific PCR primers on the analysis of dark fermentation hydrogen-producing bacterial community. Int J Hydrogen Energy. 2008;33(5):1586–1592.

[233] Chang -J-J, Chen W-E, Shih S-Y, et al. Molecular detection of the clostridia in an anaerobic biohydrogen fermentation system by hydrogenase mRNA-targeted reverse transcription-PCR. Appl Microbiol Biotechnol. 2006;70(5):598–604. 16217655 10.1007/s00253-005-0106-7

[234] Chang -J-J, WU J-H, WEN F-S, et al. Molecular monitoring of microbes in a continuous hydrogen-producing system with different hydraulic retention time. Int J Hydrogen Energy. 2008;33(5):1579–1585.

[235] Tolvanen KE, Koskinen PE, Ylikoski AI, et al. Quantitative monitoring of a hydrogen-producing Clostridium butyricum strain from a continuous-flow, mixed culture bioreactor employing real-time PCR. Int J Hydrogen Energy. 2008;33(2):542–549.

[236] Fuess LT, Júnior ADNF, Machado CB, et al. Temporal dynamics and metabolic correlation between lactate-producing and hydrogen-producing bacteria in sugarcane vinasse dark fermentation: the key role of lactate. Bioresour Technol. 2018;247:426–433. 28965073 10.1016/j.biortech.2017.09.121

[237] KIM D-H, Jang S, Yun Y-M, et al. Effect of acid-pretreatment on hydrogen fermentation of food waste: microbial community analysis by next generation sequencing. Int J Hydrogen Energy. 2014;39(29):16302–16309.

[238] Favaro L, Alibardi L, Lavagnolo MC, et al. Effects of inoculum and indigenous microflora on hydrogen production from the organic fraction of municipal solid waste. Int J Hydrogen Energy. 2013;38(27):11774–11779.

[239] Guo XM, Trably E, Latrille E, et al. Hydrogen production from agricultural waste by dark fermentation: a review. Int J Hydrogen Energy. 2010;35(19):10660–10673.

[240] Valdez-vazquez I, Poggi-varaldo HM. Hydrogen production by fermentative consortia. Renew Sust Energ Rev. 2009;13(5):1000–1013.

[241] Crow DR. Principles and applications of electrochemistry. Florida, USA: Routledge; 1994.

[242] Lalman JA, Bagley DM. Anaerobic degradation and methanogenic inhibitory effects of oleic and stearic acids. Water Res. 2001;35:2975–2983. 11471698 10.1016/s0043-1354(00)00593-5

[243] Saady NMC. Homoacetogenesis during hydrogen production by mixed cultures dark fermentation: unresolved challenge. Int J Hydrogen Energy. 2013;38(30):13172–13191.

[244] Diekert G, Wohlfarth G. Metabolism of homoacetogens. Antonie Van Leeuwenhoek. 1994;66(1–3):209–221. 7747932 10.1007/BF00871640

[245] Dhar BR, Elbeshbishy E, Nakhla G. Influence of iron on sulfide inhibition in dark biohydrogen fermentation. Bioresour Technol. 2012;126:123–130. 23073098 10.1016/j.biortech.2012.09.043

[246] Mohan SV, Mohanakrishna G, Goud RK, et al. Acidogenic fermentation of vegetable based market waste to harness biohydrogen with simultaneous stabilization. Bioresour Technol. 2009;100(12):3061–3068. 19230653 10.1016/j.biortech.2008.12.059

[247] KIM M-S, LEE D-Y. Fermentative hydrogen production from tofu-processing waste and anaerobic digester sludge using microbial consortium. Bioresour Technol. 2010;101(1):S48–S52. 19394818 10.1016/j.biortech.2009.03.040

[248] Braun R.Potential of Co-digestion. Rep IEA Bioenergy Task; 2002. (Report no. 37).

[249] Luste S, Luostarinen S. Anaerobic co-digestion of meat-processing by-products and sewage sludge–Effect of hygienization and organic loading rate. Bioresour Technol. 2010;101(8):2657–2664. 19931450 10.1016/j.biortech.2009.10.071

[250] KIM S, Choi K, KIM J-O, et al. Biological hydrogen production by anaerobic digestion of food waste and sewage sludge treated using various pretreatment technologies. Biodegradation. 2013;24(6):753–764. 23389715 10.1007/s10532-013-9623-8

[251] Chong M-L, Sabaratnam V, Shirai Y, et al. Biohydrogen production from biomass and industrial wastes by dark fermentation. Int J Hydrogen Energy. 2009a;34(8):3277–3287.

[252] Sinha P, Pandey A. An evaluative report and challenges for fermentative biohydrogen production. Int J Hydrogen Energy. 2011;36(13):7460–7478.

[253] LI C, Fang HH. Inhibition of heavy metals on fermentative hydrogen production by granular sludge. Chemosphere. 2007;67(4):668–673. 17182079 10.1016/j.chemosphere.2006.11.005

[254] BAO M, SU H, TAN T. Dark fermentative bio-hydrogen production: effects of substrate pre-treatment and addition of metal ions or L-cysteine. Fuel. 2013;112:38–44.

[255] Angenent LT, Karim K, Al-dahhan MH, et al. Production of bioenergy and biochemicals from industrial and agricultural wastewater. Trends Biotechnol. 2004;22(9):477–485. 15331229 10.1016/j.tibtech.2004.07.001

[256] Dong L, Zhenhong Y, Yongming S, et al. Hydrogen production characteristics of the organic fraction of municipal solid wastes by anaerobic mixed culture fermentation. Int J Hydrogen Energy. 2009;34(2):812–820.

[257] Van Niel EW, Claassen PA, Stams AJ. Substrate and product inhibition of hydrogen production by the extreme thermophile, Caldicellulosiruptor saccharolyticus. Biotechnol Bioeng. 2003;81(3):255–262. 12474247 10.1002/bit.10463

[258] Ciranna A, Ferrari R, Santala V, et al. Inhibitory effects of substrate and soluble end products on biohydrogen production of the alkalithermophile Caloramator celer: kinetic, metabolic and transcription analyses. Int J Hydrogen Energy. 2014;39(12):6391–6401.

[259] Wong YM, WU TY, Juan JC. A review of sustainable hydrogen production using seed sludge via dark fermentation. Renew Sust Energ Rev. 2014;34:471–482.

[260] Chong M-L, Yee PL, Abd Aziz S, et al. Effects of pH, glucose and iron sulfate concentration on the yield of biohydrogen by Clostridium butyricum EB6. Int J Hydrogen Energy. 2009b;34(21):8859–8865.

[261] Millat T, Janssen H, Bahl H, et al. The pH-induced metabolic shift from acidogenesis to solventogenesis in Clostridium acetobutylicum- from experiments to models. Proceedings of the Experimental Standard Conditions of Enzyme Characterization; 2011; Rudesheim/Rhein, Germany. 2013.

[262] Varjani S, Shah AV, Vyas S, et al. Processes and prospects on valorizing solid waste for the production of valuable products employing bio-routes: a systematic review. Chemosphere. 2021;282:130954. 34082315 10.1016/j.chemosphere.2021.130954

[263] Fermoso FG, Van Hullebusch E, Collins G, et al. Trace elements in anaerobic biotechnologies. London, UK: IWA Publishing; 2019.

[264] WID N, Selaman R, Jopony M. Enhancing phosphorus recovery from different wastes by using anaerobic digestion technique. Adv Sci Lett. 2017;23(2):1437–1439.

[265] Chong CC, Cheng YW, Ishak S, et al. Anaerobic digestate as a low-cost nutrient source for sustainable microalgae cultivation: a way forward through waste valorization approach. SciTotal Environ. 2022;803:150070. 10.1016/j.scitotenv.2021.150070 34525689

[266] SHI L-D, LV P-L, Wang M, et al. A mixed consortium of methanotrophic archaea and bacteria boosts methane-dependent selenate reduction. SciTotal Environ. 2020;732:139310. 10.1016/j.scitotenv.2020.139310 32442771

[267] Devda V, Chaudhary K, Varjani S, et al. Recovery of resources from industrial wastewater employing electrochemical technologies: status, advancements and perspectives. Bioengineered. 2021;12(1):4697–4718. 34334104 10.1080/21655979.2021.1946631 PMC8806852

[268] WU J-Y, LAY C-H, Chia SR, et al. Economic potential of bioremediation using immobilized microalgae-based microbial fuel cells. Clean Technol Envir. 2021;23:2251–2264.

[269] Wang G, WEI L, CAO C, et al. Novel resolution-contrast method employed for investigating electron transfer mechanism of the mixed bacteria microbial fuel cell. Int J Hydrogen Energy. 2017;42(16):11614–11621.

[270] Bayané A, Guiot SR. Animal digestive strategies versus anaerobic digestion bioprocesses for biogas production from lignocellulosic biomass. Rev Environ Sci Bio/Technol. 2011;10(1):43–62.

[271] Barnes S, Keller J. Cellulosic waste degradation by rumen-enhanced anaerobic digestion. Water Sci Technol. 2003;48(4):155–162. 14531434

[272] Kaplan-shabtai V, Indugu N, Hennessy ML, et al. Using structural equation modeling to understand interactions between bacterial and archaeal populations and volatile fatty acid proportions in the rumen. Front Microbiol. 2021;12:1457. 10.3389/fmicb.2021.611951 PMC8248675 34220728

[273] Nguyen LN, Nguyen AQ, Johir MAH, et al. Application of rumen and anaerobic sludge microbes for bio harvesting from lignocellulosic biomass. Chemosphere. 2019;228:702–708. 31063917 10.1016/j.chemosphere.2019.04.159

[274] Takizawa S, Baba Y, Tada C, et al. Pretreatment with rumen fluid improves methane production in the anaerobic digestion of paper sludge. Waste Manage. 2018;78:379–384. 10.1016/j.wasman.2018.05.046 32559924

[275] YUE Z-B, LI -W-W, YU H-Q. Application of rumen microorganisms for anaerobic bioconversion of lignocellulosic biomass. Bioresour Technol. 2013;128:738–744. 23265823 10.1016/j.biortech.2012.11.073

[276] Zhao B-H, YUE Z-B, NI B-J, et al. Modeling anaerobic digestion of aquatic plants by rumen cultures: cattail as an example. Water Res. 2009;43:2047–2055. 19297004 10.1016/j.watres.2009.02.006

[277] HU ZH, YU HQ. Anaerobic digestion of cattail by rumen cultures. Waste Manag. 2006;26(11):1222–1228. 16198552 10.1016/j.wasman.2005.08.003

[278] Choudhury PK, Salem AZM, Jena R. Rumen microbiology: An overview. In: Puniya AK, Singh R, Kamra DN, et al., editors. Rumen microbiology: from evolution to revolution. New Delhi, India: Springer; 2015.

[279] Liang J, Fang W, Wang Q, et al. Metagenomic analysis of community, enzymes and metabolic pathways during corn straw fermentation with rumen microorganisms for volatile fatty acid production. Bioresour Technol. 2021a;342:126004. 34583109 10.1016/j.biortech.2021.126004

[280] Sirohi SK, Singh N, Dagar SS, et al. Molecular tools for deciphering the microbial community structure and diversity in rumen ecosystem. Appl Microbiol Biotechnol. 2012;95(5):1135–1154. 22782251 10.1007/s00253-012-4262-2

[281] Belanche A, Palma-hidalgo JM, Nejjam I, et al. In vit assessment of the factors that determine the activity of the rumen microbiota for further applications as inoculum. J Sci Food Agric. 2019;99(1):163–172. 29851076 10.1002/jsfa.9157

[282] Rico JL, Reardon KF, De Long SK. Inoculum microbiome composition impacts fatty acid product profile from cellulosic feedstock. Bioresour Technol. 2021;323:124532. 33422791 10.1016/j.biortech.2020.124532

[283] Boone DR, Garrity G, Castenholz RW. Bergey’s manual of systematic bacteriology: volume one: the archaea and the deeply branching and phototrophic bacteria. New York: Springer; 2011.

[284] Sakai S, Imachi H, Hanada S, et al. Methanocella paludicola gen. nov., sp. nov., a methane-producing archaeon, the first isolate of the lineage ‘Rice Cluster I’, and proposal of the new archaeal order Methanocellales ord. nov. Int J Syst Evol Microbiol. 2008;58(4):929–936. 18398197 10.1099/ijs.0.65571-0

[285] Dridi B, Fardeau M-L, Ollivier B, et al. Methanomassiliicoccus luminyensis gen. nov., sp. nov., a methanogenic archaeon isolated from human faeces. Int J Syst Evol Microbiol. 2012;62(Pt_8):1902–1907. 22859731 10.1099/ijs.0.033712-0

[286] Kurr M, Huber R, König H, et al. Methanopyrus kandleri, gen. and sp. nov. represents a novel group of hyperthermophilic methanogens, growing at 110°C. Arch Microbiol. 1991;156(4):239–247.

[287] Meile L, Abendschein P, Leisinger T. Transduction in the archaebacterium Methanobacterium thermoautotrophicum Marburg. J Bacteriol. 1990;172(6):3507–3508. 2345156 10.1128/jb.172.6.3507-3508.1990 PMC209168

[288] LUO Y, Pfister P, Leisinger T, et al. The genome of archaeal prophage ψm100 encodes the lytic enzyme responsible for autolysis of Methanothermobacter wolfeii. J Bacteriol. 2001;183(19):5788–5792. 11544247 10.1128/JB.183.19.5788-5792.2001 PMC95476

[289] Nölling J, Groffen A, De Vos WM. φ F1 and φF3, two novel virulent, archaeal phages infecting different thermophilic strains of the genus Methanobacterium. Microbiology. 1993;139:2511–2516.

[290] Chan C, LAU S, Husaini A, et al. Identification of methane-producing bacteria from palm oil mill sludge (POMS) with solid cud from ruminant stomach. J Biochem, Microbiol Biotechnol. 2014;2:23–26.

[291] Van Eerten-jansen MCAA, Veldhoen AB, Plugge CM, et al. Microbial community analysis of a methane-producing biocathode in a bioelectrochemical system. Archaea. 2013;2013:481784. 24187516 10.1155/2013/481784 PMC3800620

[292] ZHU C, Zhang J, Tang Y, et al. Diversity of methanogenic archaea in a biogas reactor fed with swine feces as the mono-substrate by mcrA analysis. Microbiol Res. 2011;166:27–35. 20116227 10.1016/j.micres.2010.01.004

[293] Luton PE, Wayne JM, Sharp RJ, et al. The mcrA gene as an alternative to 16S rRNA in the phylogenetic analysis of methanogen populations in landfillbbThe GenBank accession numbers for the mcrA sequences reported in this paper are AF414034–AF414051 (See Fig. 2) and AF414007–AF414033 (environmental isolates in Fig. 3). Microbiology. 2002;148(Pt 11):3521–3530. 12427943 10.1099/00221287-148-11-3521

[294] Ueno Y, Haruta S, Ishii M, et al. Characterization of a microorganism isolated from the effluent of hydrogen fermentation by microflora. J Biosci Bioeng. 2001;92(4):397–400. 16233118 10.1263/jbb.92.397

[295] Hung C-H, LEE K-S, Cheng L-H, et al. Quantitative analysis of a high-rate hydrogen-producing microbial community in anaerobic agitated granular sludge bed bioreactors using glucose as substrate. Appl Microbiol Biotechnol. 2007;75(3):693–701. 17440720 10.1007/s00253-007-0854-7

[296] Wang X, Hoefel D, Saint C, et al. The isolation and microbial community analysis of hydrogen producing bacteria from activated sludge. J Appl Microbiol. 2007;103(5):1415–1423. 17953552 10.1111/j.1365-2672.2007.03370.x

[297] Yanling Y, Zhenmei L, Hang M, et al. Dynamic changes of microbial community diversity in a photohydrogen producing reactor monitored by PCR-DGGE. J Environ Sci. 2008;20(9):1118–1125. 10.1016/s1001-0742(08)62158-0 19143320

[298] Cheng C-H, Hung C-H, LEE K-S, et al. Microbial community structure of a starch-feeding fermentative hydrogen production reactor operated under different incubation conditions. Int J Hydrogen Energy. 2008;33(19):5242–5249.

[299] Davila-vazquez G, De León-rodríguez A, Alatriste-mondragón F, et al. The buffer composition impacts the hydrogen production and the microbial community composition in non-axenic cultures. Biomass Bioenergy. 2011;35(7):3174–3181.

[300] Chen -C-C, Chuang Y-S, LIN C-Y, et al. Thermophilic dark fermentation of untreated rice straw using mixed cultures for hydrogen production. Int J Hydrogen Energy. 2012;37(20):15540–15546.

[301] Poleto L, Souza P, Magrini FE, et al. Selection and identification of microorganisms present in the treatment of wastewater and activated sludge to produce biohydrogen from glycerol. Int J Hydrogen Energy. 2016;41(7):4374–4381.

[302] JO JH, Jeon CO, LEE DS, et al. Process stability and microbial community structure in anaerobic hydrogen-producing microflora from food waste containing kimchi. J Biotechnol. 2007;131(3):300–308. 17716770 10.1016/j.jbiotec.2007.07.492

[303] NIU M, Liang W, Wang F. Methane biotransformation in the ocean and its effects on climate change: a review. Sci China Earth Sci. 2018;61(12):1697–1713.

